# Quantifying Arctic-boreal methane emissions using atmospheric observations and a global inverse model

**DOI:** 10.1038/s41612-026-01348-1

**Published:** 2026-02-14

**Authors:** L. S. Basso, C. Rödenbeck, V. Brovkin, G. Georgievski, M. Heimann, M. Göckede

**Affiliations:** 1 Department of Biogeochemical Signals, Max Planck Institute for Biogeochemistry, Jena, Germany; 2 Department Climate Dynamics, Max Planck Institute for Meteorology, Hamburg, Germany; 3 Earth Resilience Science Unit, Potsdam Institute for Climate Impact Research, Potsdam, Germany

**Keywords:** Climate sciences, Environmental sciences

## Abstract

The Arctic-Boreal region is vulnerable to rapid climate change. Thawing of the permafrost and extended warm periods are expected to turn the region into a hotspot of enhanced CH_4_ emissions. We estimated CH_4_ fluxes by assimilating atmospheric CH_4_ mixing ratio data from a regional network into a global atmospheric inverse model, resulting in a mean uncertainty reduction of ~68% across the domain and improved agreement with observations. From 2010 to 2021, the Arctic-Boreal region emitted 45.4 ± 0.7 TgCH_4_ y^−1^ ( ~ 7% of global emissions), with no significant overall trend. However, on the regional scale a positive trend emerged in the Western Siberia Lowlands. Arctic-Boreal wetland emissions increased during warmer years, suggesting possible future increases as warming continues. Emissions varied regionally, with western Russia showing higher fluxes. Changes in winter hydroclimate significantly influenced emissions in the Western Siberian Lowlands, likely by enhancing the availability of soil moisture through snowmelt in spring. Our findings emphasize the importance of accounting for both temperature effects and changes in wetness, when assessing Arctic CH_4_ future emissions.

## Introduction

Atmospheric methane (CH_4_) levels, the second largest contributor to climate change, have increased by approximately 2.6 times since the pre-industrial era^1^, reaching unprecedented levels with highly variable trends over time. The causes of its recent increase remain incompletely understood. This is partly due to the challenge of determining whether the upward trend is driven by anthropogenic emissions, changes in natural sources, or both. Anthropogenic emissions, primarily from agriculture, fossil fuel production and use, and waste management, are being attributed in large part to this increase^1,2^. Additionally, changes in natural sources (mainly wetland emissions) due to climate variability and change have been identified as a contributing factor^1,3,4^. The recent increase in CH_4_ levels is of particular concern because it amplifies the greenhouse effect, contributing to accelerating climate change. Therefore, it is essential to gain a better understanding on the natural and anthropogenic processes resulting in CH_4_ emissions, as well as to quantify CH_4_ sources at global and regional scales in order to reduce uncertainties in the global CH_4_ budget and its feedback with the climate system.

The Arctic region plays a significant role in the global CH_4_ budget, acting as both a potential major natural source and a sensitive indicator of climate change^5,6^. The northern permafrost region covers up to 21 million km^2^ of land in the Northern Hemisphere^7^, and the upper three meters of permafrost soils are estimated to store 1035 ± 150 Pg of soil organic carbon^8^. Wetlands and lakes in the Arctic are key contributors to CH_4_ emissions, primarily due to the anaerobic decomposition of organic matter. Temperatures in the Arctic region have risen at rates nearly four times the global average over the last few decades^9^, causing the permafrost to progressively thaw. As a consequence, the increased availability of organic matter for microbial decomposition could induce methanogenesis and release CH_4_ to the atmosphere, which could result in a positive feedback loop that contributes to further warming and climate change due to increased emissions. It is also possible that changing environmental conditions, such as changes in land surface hydrology^10^, and changes in the landscape topography, e.g., the formation of thermokarst lakes^11^, affect natural emissions and may contribute to increased CH_4_ production and loss to the atmosphere. This could be a particularly important effect in Arctic CH_4_ emissions. A comprehensive understanding of these region-specific CH_4_ emission mechanisms is therefore critical for accurately projecting feedbacks between the Arctic and the global climate system.

Although the importance of the Arctic region as a significant source of CH_4_ emissions is well established, substantial uncertainty persists in quantifying its overall contribution to the global CH_4_ budget^12^. Despite extensive research, there is still considerable disagreement between different methods for quantifying regional scale emissions, since the understanding of the greenhouse gas (GHG) balance of this region remains poorly constrained due to limited data availability (both temporal and spatial) and its heterogeneous landscape. In a recent study, Hugelius et al. ^13^ compared CH_4_ budgets derived from land-surface process modeling (bottom-up) and atmospheric inversion (top-down) approaches for the entire permafrost region over the period 2000–2017. The results indicated a large discrepancy between the two approaches. The bottom-up budget was estimated to be approximately 50 (29–71; mean upper and lower 95% CI) TgCH_4_ y^−1^, while the top-down estimate was 20 (15–24) TgCH_4_ y^-1^. With median budgets differing by a factor of ~2.5 and uncertainty ranges that barely overlap, identifying and addressing the underlying causes of this discrepancy is essential. Recently, annual mean CH_4_ emissions from vegetated wetlands north of 45°N during 2016–2022 was estimated at 22.8 ± 2.4TgCH_4_y^−1^, ranging from 15.7 ± 1.8TgCH_4_y^−1^ to 51.6 ± 2.2TgCH_4_y^−1^, depending on the wetland dataset used in the machine-learning-based upscaling approach^14^.

In this context, it is important to note that there is no clear agreement on long-term trends in Arctic-Boreal CH_4_ emissions. Studies have reported different regional patterns and trends over time. For example, Rößger et al.^15^ estimated a positive trend in CH_4_ emissions during early summer months between 2002 and 2019 from a long-term eddy covariance site in the Lena River Delta. Similarly, twenty-nine years of measurements at Barrow indicate a recent increase in emissions during late fall and early winter on the North Slope. However, the mean seasonal enhancements for July to December do not show significant trends, despite surface air temperature increases^16^. Similarly, Zhang et al.^17^ estimated an increase in Arctic wetland emissions of 0.6–0.8 TgCH_4_ y^−1^ between 2000 and 2020 (primarily during the early growing season, May-July), based on an ensemble of process-based wetland models. Yuan et al.^18^ also reported an increasing trend in wetland emissions during early summer (June-July), driven by an increase in temperature. They found that 56% of this increase occurred in the Western Siberian lowlands. Ward et al.^19^ estimated an increase trend in July-August on the North Slope of Alaska between 1986 and 2021. Liu et al.^20^ estimated a decrease in CH_4_ seasonal cycle amplitude in the northern high latitudes since 1984, mainly due to an increase in natural emissions from wetlands. However, Wittig et al.^12^ based on regional inverse modelling reported that the majority of the CH_4_ sources, currently present in the high northern latitudes are poorly constrained by the existing observation network. Only CH_4_ fluxes from wetlands are adequately constrained, predominantly in North America^12^. Between 2008 and 2019, they found that wetland emissions showed a slight negative trend in North America and a slight positive trend in East Eurasia.

**Fig. 1:**
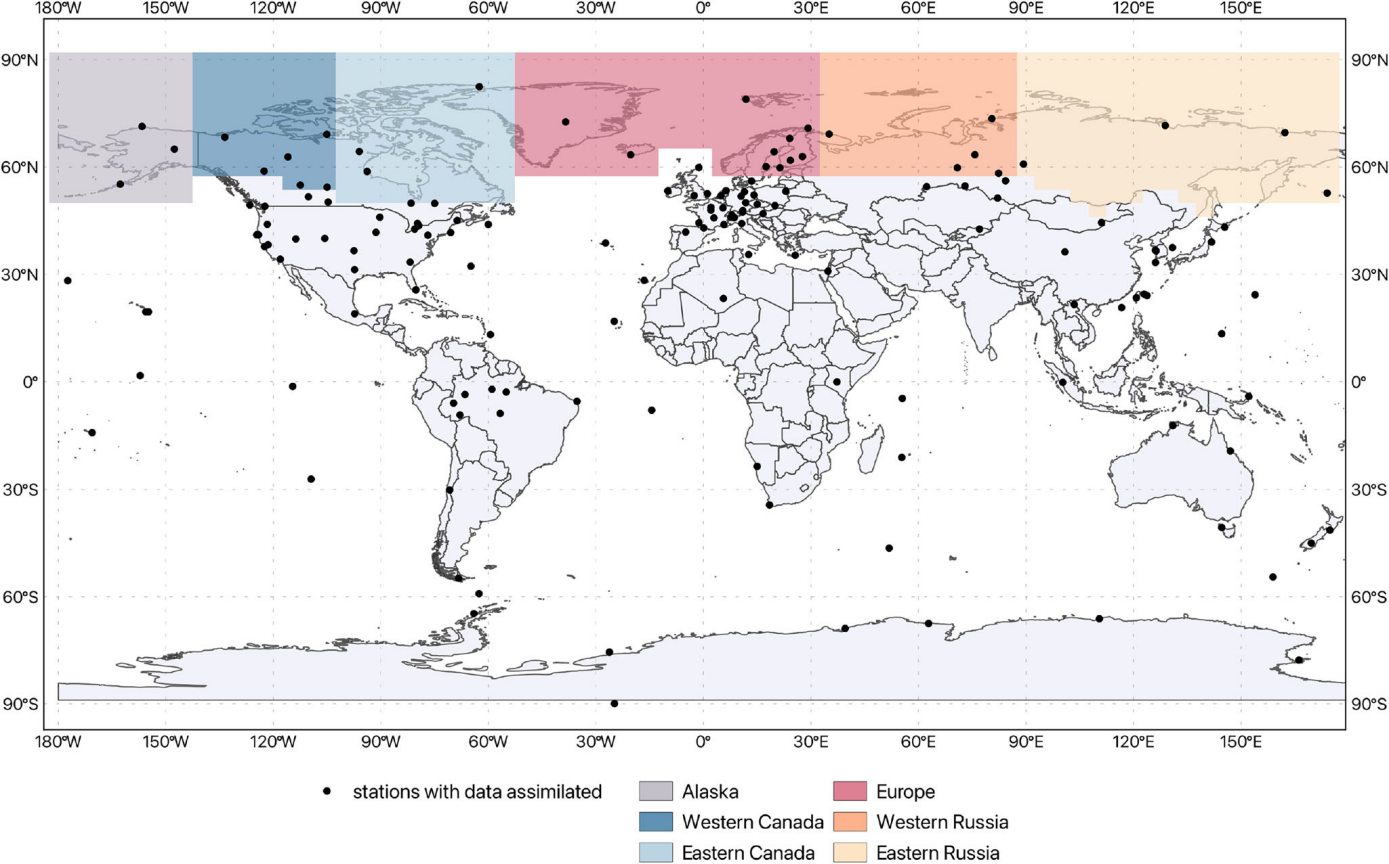
Geographic distribution of surface sites operated by different network providers where flask-based and/or continuous in-situ CH_4_ measurements are assimilated in the inverse model (black dots). The colored boxes delineate the Arctic-Boreal regions (from west to east: Alaska, western Canada, eastern Canada, Europe, western Russia, eastern Russia), as defined based on The Boreal-Arctic Wetland and Lake Dataset (BAWLD)^57^.

A comprehensive understanding of the dynamics of CH_4_ emissions in the Arctic is crucial for accurately predicting future climate scenarios and developing effective GHG emission mitigation strategies. This paper aims to estimate CH_4_ emissions in the Arctic-Boreal region over the last decade (2010–2021) using atmospheric surface observations of CH_4_ mixing ratios in global inverse modelling, with a particular emphasis on data from Arctic-Boreal regional observation networks (Fig. 1, Supplementary Fig. 1). These high-latitude monitoring networks could help to provide the spatial and temporal coverage necessary to enhance the accuracy of emission estimates in this region. These estimates are sometimes underrepresented in global datasets used for atmospheric inversions. This is due to the limited incorporation of available Arctic-Boreal data in many inversion systems, including some compiled by the Global Carbon Project (GCP). By integrating these datasets, this study quantifies CH_4_ emissions across the Arctic-Boreal region and its contribution to the global CH_4_ budget, while examining the spatial variability and seasonality in six sub-regions. We also investigate regional temporal trends in CH_4_ emissions from 2010 to 2021, allowing detection of potential changes over time. Furthermore, we analyze the relationship between CH_4_ fluxes and key environmental drivers, including air temperature, total precipitation, and snow depth for the Arctic-Boreal region and at per grid-cell level. This comprehensive approach provides a more detailed understanding of CH_4_ emissions in this region and their implications in the context of global climate change.

## Results and discussion

### Mean annual CH_4_ emissions and regional variability

We estimated Arctic-Boreal CH_4_ emissions using the Jena CarboScope Global Atmospheric Inversion System^21^, which is a linear Bayesian framework that optimizes surface-atmosphere fluxes by combining prior flux estimates with atmospheric CH_4_ observations, while accounting for their respective uncertainties. The inversions were carried out with a horizontal resolution of ~3.8° latitude x 5° longitude, and assimilated in situ CH_4_ observations from 154 stations worldwide, including 33 in the Arctic-Boreal region for the period between 2010 and 2021. The posterior global annual mean net CH_4_ emission resulting from the global inversion is 654.8 TgCH_4_ y^−1^, compared to a prior global annual mean net CH_4_ emission of 610.9 TgCH_4_ y^−1^ corresponding to a net global adjustment of 43.9 TgCH_4_ y^−1^. Due to the relatively coarse resolution and the presence of overlapping CH_4_ sources and sinks within a grid cell, any deviations from prior flux estimates were attributed to the relevant processes in proportion to their respective uncertainties. The inversion optimizes CH_4_ emissions globally. This means that the posterior mole fractions over the Arctic-Boreal region reflect local emission adjustments as well as emission corrections in other regions that influence Arctic concentrations through large-scale atmospheric transport. To evaluate how well the inversion fitted the assimilated Arctic-Boreal data, we compared the prior and posterior mole fractions with the observations for all stations with assimilated data (see Supplementary Table 1). The inversion substantially reduced the mean model-observation difference and increased the correlation between the posterior mole fractions and the observations, demonstrating improved model performance.

**Fig. 2:**
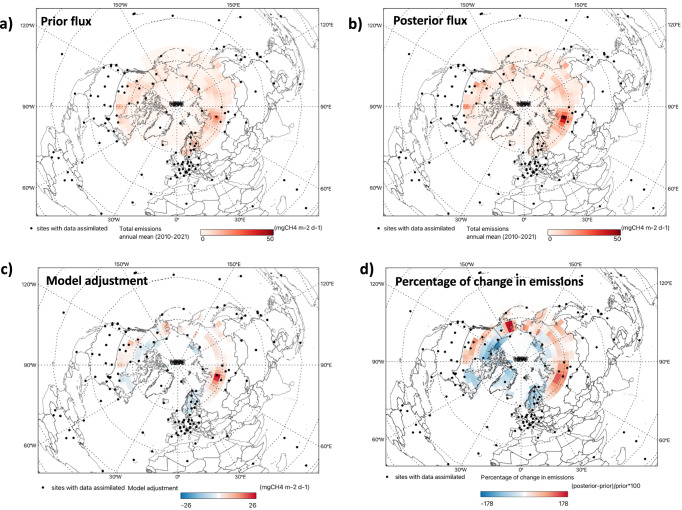
Spatial patterns of prior and posterior Arctic-Boreal CH_4_ flux estimates. Annual mean prior (**a**) and posterior (**b**) CH_4_ flux estimates for the Arctic-Boreal region (2010–2021), as well as the difference (**c**) between posterior and prior fluxes. **d** shows the spatial pattern of percentage changes from prior to posterior estimates, calculated as (posterior-prior)/prior*100%. Positive values indicate regions where prior estimates underestimated emissions compared with posterior estimates, while negative values represent areas where prior emissions overestimate CH_4_ emissions compared with the posterior estimates.

**Fig. 3:**
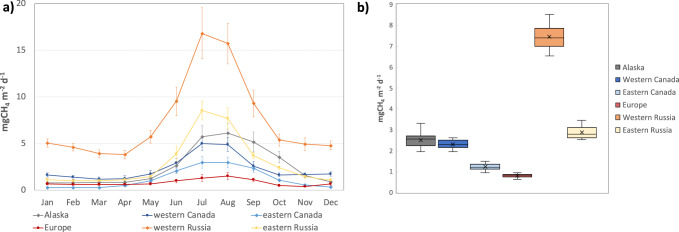
Monthly and annual mean total CH_4_ fluxes across Arctic-Boreal sub-regions. **a** The 12-year monthly mean (2010–2021) total CH_4_ fluxes for each of the sub-regions (excluding exclusively ocean grid-cells), error bars represented the monthly mean standard deviation (1 sigma). **b** the annual mean of total emissions for each of the sub-regions (excluding exclusively ocean grid-cells).

Our atmospheric inversions indicate that the Arctic-Boreal region is a source of CH_4_ to the atmosphere, with a posterior mean total annual emission of 45.4 ± 0.7 TgCH_4_ y^−1^ (averaged over 2010-2021). This represents a ~ 16% (6.4 TgCH_4_ y^−1^) increase relative to prior estimates, suggesting that the prior fluxes underestimate CH_4_ emissions (Table 1). The posterior flux estimates exhibit a distinct spatial variability (Fig. 2) across the six sub-regions of the Arctic-Boreal domain (Fig. 1; Alaska, western Canada, eastern Canada, Europe, western Russia and eastern Russia). To enable a more comprehensive comparison with other studies, Table 2 and Fig. 3b summarize the mean annual total emissions for all land and coastal regions; only ocean grid cells are excluded. Due to the model’s coarse resolution ( ~ 3.8° latitude and 5° longitude) grid-cells with both land and ocean areas are included. The regions showed substantial differences in emission magnitudes. Western Russia dominates regional emissions, with total CH_4_ fluxes two to six times higher than those observed in other regions, mainly due to higher emissions from wetlands, anthropogenic sources and other natural sources (Table 2).

**Table 1:** Mean annual prior (bottom-up model or inventory-based estimates before data assimilation) and posterior (adjusted estimates after the inversion with assimilation of observational data) CH_4_ emissions from 2010 to 2021 per category for the Arctic-Boreal region

Categories	Prior (TgCH_4_ y^−1^)	Posterior (TgCH_4_ y^−1^)	Reference (prior flux)
Wetlands	18.9 ± 2.1	21.5 ± 0.8	JSBACH model^44^
Anthropogenic	9.3 ± 0.6	11.6 ± 0.5	EDGAR v.8^47^
Other natural	6.1 ± 0.3	7.9 ± 0.3	Termites and herbivore (JSBACH)^44^; and natural geological^48^
Fire	2.8 ± 0.4	2.4 ± 0.3	GCP^1^
Oceans	2.0 ± 0.1	2.0 ± 0.1	Weber et al.^49^
Total	39.0 ± 2.3	45.4 ± 0.7

**Table 2:** Mean annual total CH_4_ emissions from 2010 to 2021 for each region represented by the colored boxes in Fig. 1 (excluding grid-cells exclusively covered by ocean, therefore the totals differ from those in Table 1), calculated from prior and posterior estimates (and absolute uncertainties of the prior and posterior fluxes, see Methods “Model Setup” section for details)

Region	Prior (TgCH_4_ y^−1^)	Posterior (TgCH_4_ y^−1^)	Prior (mgCH_4_ m^−2^ d^−1^)	Posterior (mgCH_4_ m^−2^ d^−1^)
Alaska	2.0 ± 0.2	3.0 ± 0.1	1.7 ± 0.08	2.5 ± 0.05
western Canada	4.6 ± 0.6	4.4 ± 0.2	2.7 ± 0.35	2.3 ± 0.14
eastern Canada	5.7 ± 1.1	5.0 ± 0.4	1.5 ± 0.38	1.2 ± 0.15
Europe	4.3 ± 0.5	2.3 ± 0.2	1.5 ± 0.15	0.8 ± 0.06
western Russia	10.9 ± 1.2	16.9 ± 0.5	5.0 ± 0.52	7.5 ± 0.22
eastern Russia	11.2 ± 0.9	13.5 ± 0.5	2.6 ± 0.18	2.9 ± 0.10
Arctic-Boreal	38.7 ± 2.3	45.0 ± 0.7	2.5 ± 0.13	2.9 ± 0.04

The model adjustments are spatially heterogeneous: western Russia exhibits the largest model adjustment ( + 55%), followed by Alaska ( + 48%) and eastern Russia ( + 20%; Fig. 2, Table 2, Supplementary Fig. 2). In contrast, Europe shows the largest reduction (-46%), followed by eastern Canada (−13%) and western Canada (-6%). Note that in this study, “Europe” refers only to the region defined in Fig. 1. While both Russia and Alaska exhibit positive posterior increments, the absolute increase in western Russia ( ~ 6 TgCH_4_ y^−1^) far exceeds that in Alaska ( ~ 1 TgCH_4_ y^−1^; Table 2), indicating that western Russia is the primary driver of the net Arctic-Boreal CH_4_ increase. Posterior adjustments increase the importance of eastern Russia in terms of flux per unit area, although uncertainties remain high due to sparse observational constraints. Across the domain, the mean uncertainty reduction (UR) for 2010-2021 is 68%, with the highest URs in eastern and western Canada, and Europe (61%), followed by western Russia (59%), eastern Russia (43%), and Alaska (42%). Validation of prior and posterior mole fractions against independent mole fractions observations from four non-assimilated sites shows consistent reduction in posterior bias and improved correlations at most stations, except at Yakutsk (YAK) in eastern Russia (Supplementary Table 2). At this location, correlation improves only slightly, reflecting the limited observational constraints, despite a substantial decrease in model-observation mismatch.

Wetlands were the primary source of CH_4_ to the atmosphere (47% of the total posterior flux); followed by anthropogenic sources (26%), other natural sources (17%), fire (5%) and oceans (4%). Our inversion estimates a total net posterior global CH_4_ emission of 654.8 TgCH_4_ y^−1^, of which the Arctic-Boreal region contributes ~7%. Of this total, Arctic-Boreal wetland CH_4_ emissions (21.5 ± 0.8 TgCH_4_ y^−1^) represent ~13% of our global wetland CH_4_ emissions (166.2 TgCH_4_ y^−1^). This highlights their significant role in the global CH_4_ cycle. A detailed comparison of these budget estimates with top-down and bottom-up reference studies is provided in section “Comparison to top-down and bottom-up estimates”.

The higher emissions in western Russia are mainly attributed to the larger wetlands and anthropogenic emissions in the region. This is consistent with previous findings^22^ that the highest CH_4_ emissions in Russia are concentrated over wetlands and oil/gas extraction areas in western Siberia. This region includes the world's largest high latitude wetland, the Western Siberian Lowlands (WSL)^23^, which accounts for 68% of western Russian wetland posterior emissions and is a major wetland CH_4_ hotspot in the Arctic-Boreal region. It accounts for 23% of the Arctic-Boreal wetland posterior emissions estimated in this study, with regional annual emissions of 9.3 ± 0.4 TgCH_4_ y^−1^ (equivalent to 16.9 ± 0.8 mgCH_4_ m^−2^ d^−1^), of which 52% comes from wetlands, 33% from anthropogenic emissions and the remaining from the other sources. Another important wetland CH_4_ hotspot is the Hudson Bay Lowlands (HBL) in eastern Canada, which accounts for 57% of eastern Canadian posterior wetland CH_4_ emissions, emitting 2.4 ± 0.3 TgCH_4_ y^−1^ (equivalent to 7.6 ± 1.4 mgCH_4_ m^−2^ d^−1^), with wetlands accounting for 92% of the total emissions. Previous inverse modelling estimates yielded an annual mean emission for HBL in the range of 2.4 to 3.4 TgCH_4_ y^−1^ (equivalent to 4.4 to 6.3 mgCH_4_ m^−2^ d^−1^, based on the domain reported on these studies)^24,25^ and 19.3 to 19.9 TgCH_4_ y^−1^ (equivalent to 10.7 to 11.0 mgCH_4_ m^−2^ d^−1^, based on the domain reported on these studies) at WSL^25^. The discrepancies in emissions in comparison to the results reported in this study could be partly related to differences in the domains considered in each study. This is mainly important in the case of WSL, where our study domain is smaller than the WSL domain from Thompson et al.^25^ (50–75°N, 60–95°E). The region south of 61°N was not included in our domain (61.2–72.7°N, 62.5–87.5°E). While for HBL our domain (49.8–57.4°N, 77.5–92.5°W) is smaller than the domains used by Thompson et al.^25^ and Miller et al.^24^ (50–60°N, 75–96°W).

### Seasonal variability in Arctic-boreal CH_4_ emissions

Analysis of CH_4_ emission estimates also shows a consistent seasonal pattern across the six Arctic-Boreal regions, with peaks generally occurring in July-August (Fig. 3). This seasonal pattern is primarily due to emissions from wetlands, which are highest in summer (July-August; 6.4 TgCH_4_ month^−1;^ accounting for 70% of total emissions during this period), followed by spring (May-June; 2.0 TgCH_4_ month^−1^; 53%) and fall (September-November; 1.4 TgCH_4_ month^−1^; 39%), with the lowest in winter (December-April; 0.1 TgCH_4_ month^−1^; 7%). The timing of the peak in wetland emissions aligns with previous bottom-up estimates^14^. Wetlands are the dominant source of CH_4_ emissions (33–78% of total annual emissions) in most regions except in Europe (17%), where anthropogenic emissions dominated (72% of total annual emissions). Anthropogenic emissions are also a large source of CH_4_ in western Russia (38% of total annual emissions), western Canada (20%) and eastern Russia (15%), while other natural emissions significantly contribute in western Canada (33% of total emissions), eastern Russia (21%) and western Russia (16%). Fire emissions are an important source in eastern Russia (10% of total emissions) and western Canada (11%). In general, the assimilation of atmospheric observational data mostly increases fluxes during the growing season, except for Europe and Canada, where prior fluxes overestimate emissions during this period. Winter fluxes increased in western Russia and western Canada, suggesting that prior fluxes underestimated anthropogenic emissions (Supplementary Fig. 3). In general, the inversion results in minor changes to the CH_4_ seasonal cycle. The timing of peak fluxes remains similar, with most adjustments affecting the magnitude rather than the phase of seasonal variability (see Supplementary Fig. 3).

### Inter-annual variability in regional CH_4_ emissions and its drivers

**Fig. 4:**
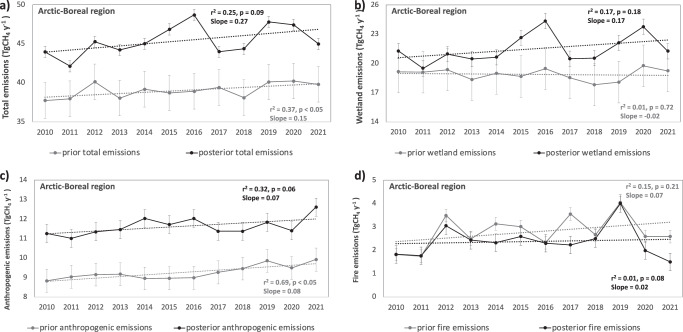
Annual mean Arctic-Boreal CH_4_ emissions. The annual mean CH_4_ emissions for the Arctic-Boreal domain (2010–2021) are displayed as (**a**) total emissions, **b** wetland emissions, **c** anthropogenic emissions, and **d** fire emissions. Error bars represent the prior and posterior annual uncertainties calculated as described in “Methods” section “Model setup”. Each panel shows the prior (grey line) and posterior (black line) estimates of emissions, along with their respective linear regression trends over time.

Arctic-Boreal total CH_4_ emissions estimates show pronounced peaks in 2016, 2019 and 2020, with annual anomalies exceeding the twelve-year mean by ∼ 2–3 TgCH_4_ y^−1^ ( ∼ 4-7%, Fig. 4). The 2016 and 2020 peaks are primarily driven by variations in wetland emissions, the dominant source of IAV in total emissions (Fig. 4b). In particular, these oscillations are largely associated with emissions from western Russia (1.5 and 1.2 TgCH_4_ y^−1^ larger than the mean, respectively) and eastern Canada (0.7 and 1.2 TgCH_4_ y^−1^ larger than the mean, respectively), regions characterised by extensive wetlands (WSL and HBL). These elevated emissions were also captured by a machine-learning-based upscaling approach for CH_4_ fluxes, with the largest interannual variations coming from Western Siberia^14^. In addition, fire emissions, mainly from eastern Russia (1.5 TgCH_4_ y^−1^ larger than the mean), contribute to this variability, particularly in 2019, further increasing CH_4_ fluxes in this year (Fig. 4d). The IAV estimated in our total emissions are consistent with those observed in other global inversions, such as CarbonTracker-CH_4_^26^ and CAMS^27^, which also show higher total CH_4_ emissions in 2016 and 2020. The 2019 anomaly in our inversion is primarily attributed to increased emissions from eastern Russia, a region with limited surface observation data, which limits the ability of the inversion to correct potential biases in the priors.

The analysis of total annual posterior emissions in the Arctic-Boreal region shows an increasing trend over time (r^2^ = 0.25, slope = 0.27 TgCH_4_ y^−1^, *p*-value = 0.09) though it is not statistically significant. According to our estimates, the anthropogenic emissions contribute substantially to the observed increase in total CH_4_ emissions over time (r^2^ = 0.32, slope = 0.07 TgCH_4_ y^−1^, *p*-value = 0.06); however, these trends are not statistically significant (Fig. 4b). According to our estimates, the spikes in anthropogenic emissions in 2014 and 2016 originate from western Russia, where anthropogenic and wetland sources are spatially overlapped. Given the coarse spatial resolution of the inversion and the shared spatial patterns of these sectors, some of the IAV attributed to anthropogenic emissions may actually be due to wetland-related processes. Future studies that incorporate isotopic or additional co-emitted tracers could help to distinguish more clearly between overlapping source contributions within inverse modelling frameworks. Posterior trends in wetland fluxes and fire emissions, though showing higher (wetlands, Fig. 4c) or comparable (fire, Fig. 4d) average slopes, were not found to be statistically significant linked to the pronounced interannual variability.

According to our estimates, emissions in Russia, mainly eastern Russia (see Supplementary Figs. 4 and 5) are primarily responsible for the observed increase in total CH_4_ emissions over time. This increase is largely driven by rising anthropogenic emissions (r² = 0.92, slope = 0.17 TgCH_4_ y^−1^, *p*-value ≤ 0.05). However, the prior anthropogenic fluxes exhibit a stronger positive trend compared to the posterior estimates (r² = 0.95, slope = 0.15 TgCH_4_ y^−1^, *p*-value ≤ 0.05; Supplementary Fig. 5). As the anthropogenic prior used in the inversion includes all source types without sectoral attribution, no distinction can be made between the sectors contributing to the observed IAV and trend in the anthropogenic prior flux. Although posterior emissions show an increasing trend, the magnitude of this trend may still be influenced by the bottom-up priors used in the inversion. Limited observational coverage in eastern Russia (see section “Sensitivity tests” for more information), in particular, reduces the ability of the inversion to correct potential biases in the priors, which can lead to over- or underestimation of CH_4_ emissions. Recent studies show that Eastern Siberia, where oil and gas industries are rapidly expanding, exhibits highly variable and increasing methane emissions linked to intensified fossil fuel activity^22^.

Although no statistically significant trend over time was estimated at a pan-Arctic scale in the wetland posterior fluxes, an analysis of climate conditions across the study region was performed to investigate the IAV drivers in Arctic-Boreal CH_4_ wetland emissions, with a focus on key environmental factors (temperature, total precipitation, and snow depth). Our results indicate that, at the pan-Arctic scale, mean annual air temperature is significantly correlated with mean annual posterior wetland CH_4_ fluxes, with higher temperatures associated with increased CH_4_ emissions (r^2^ = 0.44, slope = 2.13 TgCH_4_ y^−1^ per °C, *p*-value ≤ 0.05; Supplementary Fig. 6). At the same time, no significant linear correlations were identified between the annual mean posterior wetland fluxes and the annual total precipitation or annual mean snow depth at the scale of the Arctic-Boreal, indicating that the IAV of CH_4_ emissions could not be explained by these climate drivers at the annual scale. The temperature relationship in the posterior fluxes is most apparent during the late growing season (mean of July-September), where a significant correlation was found between the mean air temperature and mean posterior CH_4_ fluxes for the same period (r^2^ = 0.63, slope = 0.89 TgCH_4_ month^−1^ per °C, *p*-value ≤ 0.05; Supplementary Fig. 6). It is important to highlight that no significant correlation was observed between the annual mean prior wetland flux and mean air temperature, and a weaker correlation in comparison with the posterior was observed for the late growing season mean period (r^2^ = 0.09, slope = 0.39 TgCH_4_ y^−1^ per °C, *p*-value = 0.35, and r^2^ = 0.39, slope = 0.23 TgCH_4_ month^−1^ per °C, *p*-value ≤ 0.05, respectively). This suggests that the response of wetland CH_4_ emissions to a variation in air temperature from year to year is underestimated in the prior emissions. Our analysis shows a significant linearly increasing trend in annual mean temperature from 1980 to 2021 (r² = 0.62, slope = 0.6 °C y^−1^, *p* ≤ 0.05). This warming trend obviously played a major role in corresponding increases in CH_4_ emissions, also indicating that CH_4_ emissions in this region may increase over time, as the annual mean air temperature is forecast to continue increasing over the coming decades to centuries.

### Sub regional patterns in CH_4_ emissions: Western Siberian Lowland case study

The correlation analysis between CH_4_ fluxes IAV and climate conditions was also done in a more detailed version at the grid-cell level. At the grid-cell level, we identified a moderate, statistically significant increase in total annual CH_4_ emissions (r² = 0.42, slope = 0.18 TgCH_4_ y^−1^, *p*-value ≤ 0.05) in the WSL, primarily due to wetland emissions (r² = 0.45, slope = 0.17 TgCH_4_ y^−1^, *p*-value ≤ 0.05, see Supplementary Fig. 7). This trend was not observed in prior fluxes (wetland prior flux: r² = 0.07, slope = 0.02 TgCH_4_ y^−1^, *p*-value = 0.39). This contrast highlights the value of the inversion, which is based on observations and more accurately captures the IAV and changes in CH_4_ emissions in this region over time. These temporal patterns appear to be underrepresented in the bottom-up model estimates used for prior fluxes of wetlands, which show lower IAV and no change over time. Due to substantial IAV in CH_4_ fluxes estimated for this region, a longer observational record is necessary to determine if this is a sustained long-term trend or primarily a reflection of short-term variability.

**Fig. 5:**
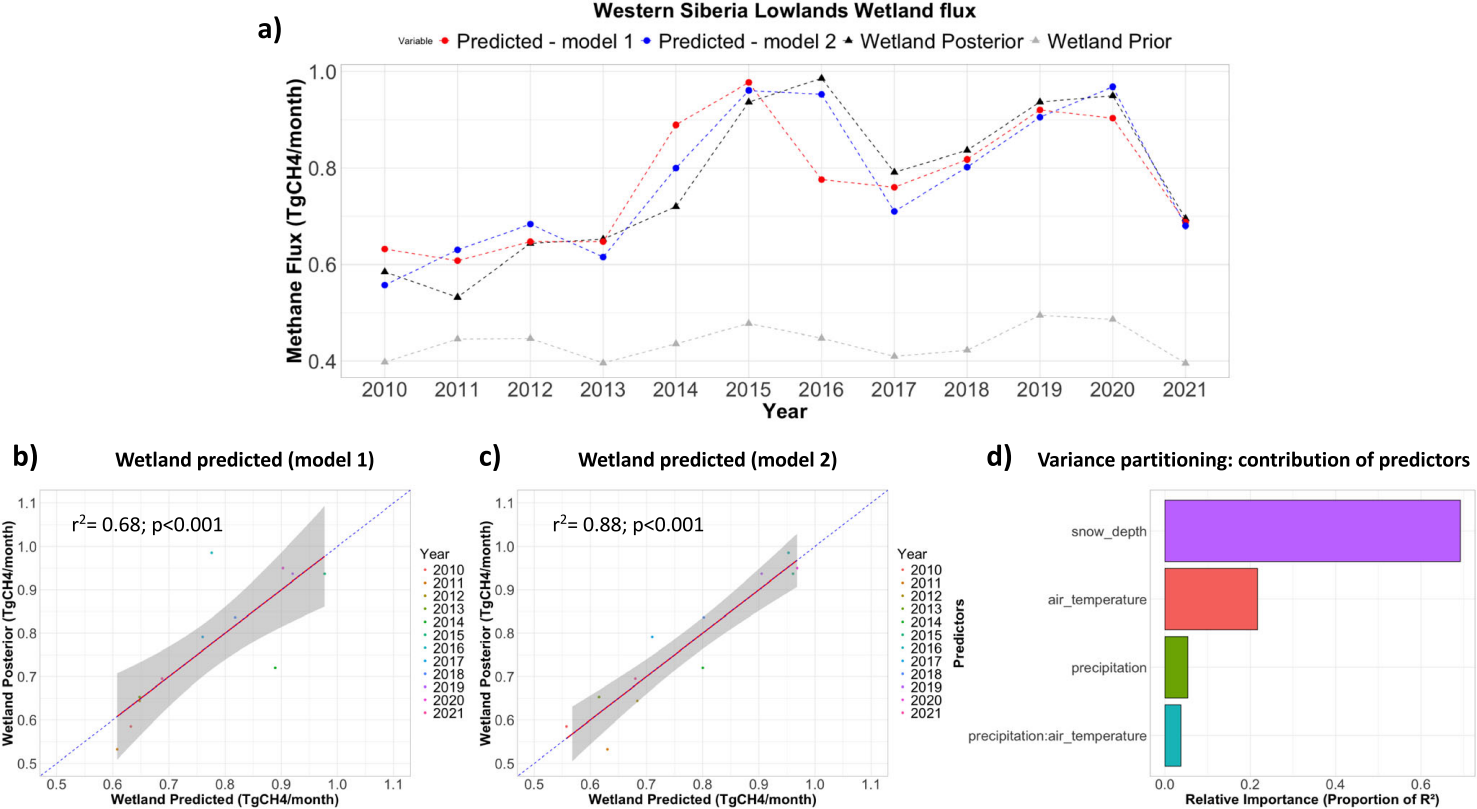
Western Siberian Lowlands wetland CH_4_ emissions. **a** Mean CH_4_ wetland emissions during the warm period (May-October) for the Western Siberian Lowlands (WSL) are displayed as posterior (black) and prior (grey) emissions, predicted emissions using a linear regression with late-winter (March-April) snow depth (named model 1, red), and predicted emissions using a multi-linear regression with late-winter (March-April) snow depth and mean air temperature and total precipitation during the growing season (May-October), including their interaction (CH_4_ emissions ~ snow depth + precipitation * air temperature) (named model 2, blue). **b**, **c** show the correlation between the predicted wetland emissions based on models 1 and 2 and posterior wetland emissions. **d** shows the contribution of each of the predictor in model 2, as by the variance partitioning analysis.

A per grid-cell linear regression analysis using the mean values for each year during July-September period revealed that the relationship between air temperature and emissions varies across the Arctic-Boreal region (Supplementary Fig. 8). While the correlations between temperature and emissions are dispersed over various areas, the highest emission hotspots (WSL) are not characterized by a particularly strong correlation. This suggests that, while temperature influences emissions across the region as a whole, other local factors may play a more dominant role in driving emissions within this specific area. For instance, in the WSL, while IAV of mean wetland CH_4_ fluxes did not show a significant correlation with mean annual air temperature (r^2^ = 0.25, slope = 0.29 TgCH_4_ y^−1^ per °C, *p*-value = 0.10), posterior wetland fluxes during the warmer period (mean value for each year during May-October period) showed a strong linear correlation with snow depth from the previous winter (mean value for each year during March-April period; r² = 0.68, slope = 3.7 TgCH_4_ month^−1^ per meter of equivalent water, p ≤ 0.05). This suggests that larger snow depths could lead to increased CH_4_ emissions. Furthermore, a multi-linear correlation was performed using warming period mean (mean of May-October) air temperature and total precipitation, along with the mean snow depth from the previous winter (mean of March-April). The multilinear model included an interaction term between temperature and precipitation, accounting for the possibility that one variable’s effect on CH_4_ emissions depends on the level of the other. This combination of climate conditions explains 88% of the WSL posterior wetland IAV (*p* ≤ 0.05, Fig. 5c). Snow depth emerged as the dominant predictor, suggesting that winter conditions may strongly influence CH_4_ emissions in this region, possibly through enhanced soil moisture availability following snowmelt. While our analysis suggests that winter conditions could be an important driver of CH_4_ emissions in this region, considering the coarse resolution and limitation of the inverse model estimates, further studies are needed to investigate this possible relationship. The same analysis was performed for the prior wetland fluxes and showed a weaker correlation in comparison with the posterior fluxes (r^2^ = 0.68, *p*-value = 0.06), suggesting that winter conditions have a larger effect on CH_4_ emissions over the warm period than reflected in the prior fluxes (from bottom-up model estimate). In the HBL region, no significant correlation was found with mean snow depth and mean air temperature, but mean wetland fluxes during May-October showed a correlation with the mean total precipitation (r^2^ = 0.27, slope = 139.6 TgCH_4_ month^−1^ per meter of precipitation per day, *p*-value = 0.08; Supplementary Fig. 9), although marginally significant. A significant relationship was observed in the mean prior fluxes though with a lower average slope (r^2^ = 0.60, slope = 112.6 TgCH_4_ month^−1^ per meter of precipitation per day, *p*-value ≤ 0.05). The discrepancy between the stronger precipitation-flux relationship in the bottom-up emissions used as priors and the moderate, marginally statistically significant relationship in the posterior fluxes could be due to differences in estimated IAV. The larger IAV in the posterior compared to the prior suggests that additional environmental factors contribute to the IAV of CH_4_ fluxes, and that the prior fluxes may therefore overestimate the relationship with precipitation. Nevertheless, although IAV is larger in the posterior fluxes, its overall magnitude in the HBL region is lower than in the WSL, which may make it difficult to identify potential drivers, given the spatial and temporal limitations of inverse modelling.

We hypothesize that the differences in climate drivers of WSL and HBL could be related to the different characteristics of these regions, as WSL have higher snow depth and larger IAV in both snow depth and CH_4_ fluxes than HBL (Supplementary Fig. 9), whereas the HBL is characterized by higher mean air temperatures, greater precipitation, and lower flux variability. These findings suggest that, although the primary climatic drivers differ between the two regions, wetness may play a key role in regulating the IAV in wetland CH_4_ emissions in both regions. At the same time, regional characteristics could have a strong influence on the exact mechanism and magnitude of the wetness effects on CH_4_ emission patterns, highlighting that explaining CH_4_ variability across the pan-Arctic domain may be challenging with a single, uniform model. We acknowledge that the spatial resolution of the inversion limits the ability to capture fine-scale heterogeneity and localised emission processes fully. In addition, the relatively low magnitude of emissions and their IAV in the HBL region compared to the WSL region may further constrain the identification of the key drivers of CH_4_ variability. To better understand the mechanisms driving CH_4_ emissions and how regional characteristics influence their variability, additional studies using higher-resolution inversions, process-based modelling and expanded in-situ and remote sensing observations are crucial.

The multi-linear correlations combining mean warmer period air temperature and total precipitation (mean of May-October) and/or mean snow depth (mean of March-April) were performed at a grid-cell basis level for the entire Arctic-Boreal domain, but statistically significant correlation was observed only for the WSL (based on r^2^, p-value and Akaike Information Criterion–AIC^28^, see Supplementary Fig. 10). We note that the correlation strength is modest even for the WSL and that the regression models are fitted to a relatively short time series. This results in considerable level of uncertainty in the inferred relationships. The lower CH_4_ fluxes and reduced IAV estimated in the other regions made it difficult to detect significant correlations at this spatial scale.

Our WSL results align with Thompson et al.^25^, who found a positively lagged (6-month lag) correlation between soil moisture and CH_4_ flux over 2005–2013 at WSL. In addition, a recent satellite-based analysis of northern high-latitude CH_4_ emissions^29^ shows that snow water equivalent is one important driver of CH_4_ seasonality, with the timing of snowmelt being closely linked to the seasonal cycle of atmospheric methane (XCH_4_), suggesting that snow dynamics influence CH_4_ release through soil moisture and insulation effects^29^. Together, these studies are consistent with our finding that winter conditions, including snow depth, could influence growing season wetland CH_4_ emissions. However, we highlight that further work is needed to fully understand and quantify this relationship. We hypothesize that the thick winter snow could act as an insulating layer, keeping soils warmer and therefore promoting sub-soil production of CH_4_. At the same time, thawing snow packs provide a steady release of water that could help maintain the wetness over the course of the growing season. The flat topography, impermeable rock layers, and specific climate conditions of western Siberia result in low drainage efficiency, promoting the formation of extensive wetlands and interconnected water bodies. The region’s broad latitudinal extent also causes a gradual spring snowmelt. These extensive floodplains and wetlands act as natural reservoirs, absorbing and gradually releasing water. This helps to regulate river discharge, reducing flood peaks and prolonging the flooding period^30–32^. According to Zakharova et al.^31^ the initial conditions of flood runoff (soil water storage) do not differ greatly from year to year, and the rate of spring runoff depends mostly on the volume of snow storage and the melting rate. Zakharova et al.^32^ estimated that, on average, 30% (ranging from 0% to 74%) of meltwater does not reach the main rivers in the northern Western Siberia. This water is stored in the active layer of the watershed and in the permanent wetlands, where it gradually evaporates or drains out to the rivers during the summer^32^. Although this mechanism could potentially explain the observed CH_4_ flux patterns, the coarse spatial resolution of the inversion and the limited length of the available time series limit our ability to robustly quantify these relationships and to fully capture fine-scale heterogeneity and localized emission processes. Further investigation and validation through complementary studies using higher-resolution inversions, process-based modelling, and expanded in-situ and remote sensing observations are required to better understand the mechanisms driving CH_4_ emissions and how regional characteristics influence their variability.

### Sensitivity tests

As a sensitivity test to evaluate the effect of assimilating regional data on constraining Arctic-Boreal CH_4_ fluxes, we performed an additional inversion using only data from seven stations (in general considered background [BKG] stations in global inversions) within the Arctic-Boreal domain, while retaining data from all other stations assimilated in the reference run outside this domain (Supplementary Fig. 11). This resulted in only 1 station in Alaska and eastern Canada, 2 in eastern Russia, and 3 in Europe. In contrast, the reference run included a denser regional observation network, adding 2 stations in Alaska, 6 in western Canada, 4 in eastern Canada, 7 in Europe, 5 in western Russia and 2 in eastern Russia. Although the total CH_4_ emission for the Arctic-Boreal region from the sensitivity test was similar (45.4 TgCH_4_ y^−1^) to the reference run estimates, the differences between the two inversion runs are not spatially homogeneous. When comparing the estimates when assimilating the regional data to the one using only BKG stations in the domain, were observed a decrease in emissions in eastern Canada (15% decrease in total emissions), Alaska (12%), Europe (5%), and eastern Russia (5%), while an increase in western Russia (11% increase in total emissions), and western Canada (9%; Supplementary Fig. 11). These results highlight the importance of assimilating regional data to better constrain the magnitude and spatial variability of Arctic-Boreal CH_4_ emissions, and are therefore essential for understanding the underlying mechanisms and controls. In regions where data are sparse or nonexistent, such as eastern Russia, the estimated flux remains close to the prior estimate. The actual atmospheric tower network in the Arctic-Boreal region is able to constrain the emissions for the North America and part of Eurasia regions with higher confidence, while regions as eastern Russia remains not well constrained^12,33,34^. As shown by the uncertainty reduction in our estimates, Canada, Europe and Western Russia have a reduction of 59–61%, while Alaska and eastern Russia have a reduction of 42–43%. These findings also emphasize that a denser observational network in the Arctic-Boreal domain would allow to constrain CH_4_ emissions with atmospheric inversions at a higher degree of detail, and further reduce posterior flux uncertainties.

Our inversion run that used a climatological prior for wetland emissions resulted in CH_4_ flux magnitudes and IAV similar to those from the reference run. This suggests that in general, the posterior estimates are not strongly influenced by prior variability, at least in regions and periods with sufficient observational data coverage. Of the six subregions analyzed, only Alaska and eastern Russia showed significant differences in IAV between the two runs (Supplementary Fig. 12). These differences could be related to the poor observational coverage in these regions, which restricts the observational constraint on posterior fluxes, resulting in estimates closest to the prior. This is consistent with the lowest uncertainty reduction in these two regions, with 42% at Alaska and 43% at eastern Russia. To test the robustness of the relation between mean wetland posterior flux (mean of May-October) and mean snow depth (mean of March-April) in WSL, we repeated the correlation analysis using posterior fluxes from the climatological wetland prior run. This analysis also showed a strong linear relationship (r² = 0.61, slope = 2.3 TgCH_4_ month^−1^ per meter of equivalent water, p ≤ 0.05).

The posterior flux estimates for the Arctic-Boreal region, based on the four sensitivity tests (using a climatological prior for wetland emissions, and μ = 1, μ = 30 and μ = 60, as described in Methods section “Design of sensitivity tests”) indicate total CH_4_ emissions ranging from 45.1 to 46.0 TgCH_4_ y^−1^ (Supplementary Fig. 13). Emission contributions by source categories were estimated as follows: 18.9–24.2 TgCH_4_ y^−1^ from wetlands, 10.1–12.9 TgCH_4_ y^−1^ from anthropogenic, 6.7–9.5 TgCH_4_ y^−1^ from other natural, 2.1–2.6 TgCH_4_ y^−1^ from fire, and 1.9–2.1 TgCH_4_ y^−1^ from ocean emissions. At the sub-regional level, posterior total emissions ranged from 2.4 to 3.5 TgCH_4_ y^−1^ in Alaska, 4.2 to 4.8 TgCH_4_ y^-1^ in western Canada, 4.6 to 6.3 TgCH_4_ y^−1^ in eastern Canada, 2.0 to 3.4 TgCH_4_ y^−1^ in Europe, 15.2 to 17.6 TgCH_4_ y^−1^ in western Russia, and 13.0 to 14.2TgCH_4_ y^−1^ in eastern Russia. In addition, total emissions were estimated at 8.5–9.4 TgCH_4_ y^−1^ for the WSL and 2.2–2.7 TgCH_4_ y^−1^ for the HBL, respectively. Our analysis showed that increasing the prior uncertainty (μ = 1) did not significantly affect the total flux magnitude for the Arctic-Boreal region. However, it did change the spatial distribution of fluxes and the relative contribution of each source category to the total emissions. The main difference was a decrease in wetland contribution, with an increase in the contributions from anthropogenic and other natural sources. Sensitivity tests suggest that source attribution depends on the choice of prior uncertainty, which highlights its influence on how emissions are distributed among different categories. Future efforts to enhance the robustness of source attribution could benefit from inversion systems that incorporate both CH_4_ and isotopic measurements, as isotopic information can differentiate emission sources with similar spatial distributions but distinct isotopic characteristics.

Finally, in the sensitivity test using *μ* = 1 (as described in Methods Section “Design of sensitivity tests”), which is more appropriated for regions with more data availability such as WSL, the results for this region shows a significant increasing trend in total CH_4_ fluxes over time (r^2^ = 0.59, slope = 0.24 TgCH_4_ y^−1^, *p*-value ≤ 0.05). In contrast, the reference run showed a moderate and statistically significant trend (r^2^ = 0.42, slope = 0.18 TgCH_4_ y^−1^, *p*-value ≤ 0.05). This difference can be linked primarily to a reduction in emissions between 2010 and 2012 in the sensitivity run using *μ* = 1. These results suggest that the increasing trend in WSL wetland emissions, even though substantial, may still be underestimated in the reference run. In addition, the correlations between wetland posterior fluxes and environmental drivers (snow depth, temperature and precipitation) are slightly lower (r^2^ = 0.74, *p* ≤ 0.05) than in the reference run, mainly due to the smaller correlation with snow depth. Meanwhile, temperature shows a stronger influence particularly during the peak of emissions. This analysis shows that climate drivers may shift slightly in their respective weight when altering the uncertainty settings in the inversion, but their combined influence still accounts for a significant portion of the IAV in wetland emissions. A denser data coverage of atmospheric observations throughout the Arctic could help to reduce the uncertainties linked to such model setup choices, and overall strengthen the role of the atmospheric perspective in constraining CH_4_ budgets over the influence of prior flux definitions.

### Comparison to top-down and bottom-up estimates

To give context to the results, we compare them with previous estimates of Arctic-boreal CH_4_ emissions (Table 3). In general, bottom-up models estimates larger emissions than top-down estimates, as previously reported by Hugelius et al.^13^ and Saunois et al.^1^ and this can be related to several factors. Top-down approaches typically use coarse spatial resolution, limiting their ability to capture the landscape heterogeneity of this region. They also rely on sparse atmospheric observations in high-latitude regions and many of them use only a limited amount of data from this region into global inversions. Additionally, there are uncertainties in atmospheric and soil CH_4_ sinks and atmospheric transport models that are particularly challenging to quantify. Meanwhile, bottom-up estimates are also affected by biased site selection, especially in heterogeneous landscapes where flux measurements may not capture the full range of variability, underrepresentation of low-flux land cover types, and uncertainties in areal coverage^13^. The wide range of CH_4_ emission estimates in the literature is partly due to the use of different time periods, as well as inconsistent and sometimes unclear definitions of the spatial domain, which makes comparisons difficult, especially when flux units are not normalized by area. We also compare the estimates with the top-down estimates reported by Hugelius et al.^13^ and found a large discrepancy between them, which is largely due to differences in spatial domains among models. Their analysis covered a smaller land region (approximately 18.4 million km²), while this study considers a broader region (approximately 37.5 million km²), including more southern areas, partly due to the coarser resolution of the transport model used in the inversion. Additionally, their top-down estimates are based on global inversions contributing to the Global Carbon Project (GCP)^35^, which generally do not incorporate the full set of regional observational data used in this study. To provide a more consistent comparison between top-down estimates used in Hugelius et al.^13^ we compared our estimates to the top-down ensemble used in their study (estimates from Saunois et al.^35^) over our domain, and we found that emissions calculated in this study were only ~1–3 Tg CH_4_ y^−1^ higher than the top down ensemble estimates^35^ (between 2010-2017 due the data availability of Saunois et al.^35^), and are generally more similar to the top-down estimates that assimilate satellite data. These results support that most of the discrepancy from our estimates to the Hugelius et al.^13^ reported top-down estimates are from differences in spatial domain covered by the respective modeling studies.

**Table 3:** Estimates of CH_4_ emissions and details of previous studies from the Arctic-Boreal region

	Region	Type of emissions	Emissions (TgCH_4_ y^−1^)
Bottom-up approaches			
Christensen et al. ^56^	>50°N	Wetlands and tundra	20 ± 13
Yuan et al.^18^	Arctic-Boreal	Wetlands	20.3 ± 0.9
Ying et al.^14^	>45°N	Wetlands	22.8 ± 2.4
Saunois et al.^1^	>60°N	Wetlands & inland waters	24 (9 – 53)
Saunois et al.^1^	>60°N	Total emissions	38 (7 – 73)
Hugelius et al.^13^	BAWLD-RECCAP2 permafrost region	Total (includes permafrost disturbances)	50 (29 – 71)
Top-down approaches			
Saunois et al.^1^	>60°N	Wetlands & inland waters	9 (7 – 17)
Saunois et al.^1^	>60°N	Total emissions	24 (18 – 29)
Hugelius et al.^13^	BAWLD-RECCAP2 permafrost region	Ecosystem emissions (excludes anthropogenic emissions)	20 (15 – 24)
CarboScope (this study)	Arctic-Boreal^a^	Total emissions	45.4 ± 0.7
CarbonTracker^b^^26^	Arctic-Boreal^a^	Total emissions	45.2 ± 4.0
CAMS (surface)^b^^27^	Arctic-Boreal^a^	Total emissions	48.6 ± 2.7
CAMS (satellite + surface)^b^^27^	Arctic-Boreal^a^	Total emissions	48.6 ± 2.7
GCP—surface^b^^1^	Arctic-Boreal^a^	Total emissions	42.1 ± 1.3
GCP—surface + satellite^b^^1^	Arctic-Boreal^a^	Total emissions	37.8 ± 1.9

In addition, mean annual total CH_4_ emissions for our Arctic-Boreal domain, based on posterior estimates from CarboScope (this study), CarbonTracker^26^ and CAMS^27^ (from 2010 to 2021), and the ensemble from Global Carbon Project (GCP; from 2010 to 2020)^1^.

^a^Estimates calculated based on the Arctic-Boreal domain used in this study.

^b^Mean annual total CH_4_ emissions and one standard deviation (TgCH_4_ y^-1^).

**Fig. 6:**
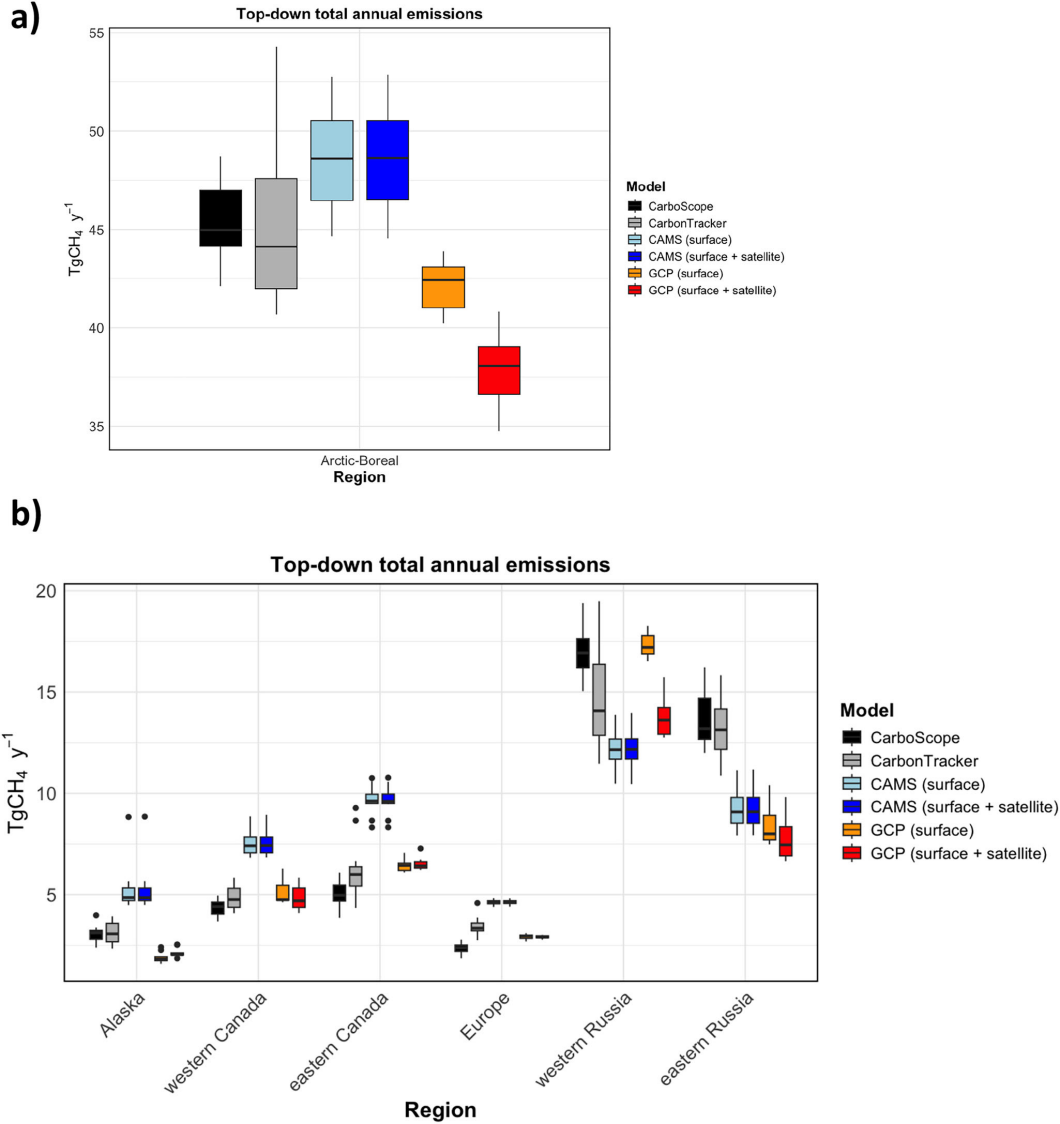
Boxplots of annual mean total CH_4_ emissions for the Arctic-Boreal region and its sub-regions. Boxplot of annual mean total emissions at **a** Arctic-Boreal region and **b** each sub-regions. Estimates are shown for CarboScope, CarbonTracker^26^, and CAMS (2010–2021)^27^, and for GCP-CH_4_^1^ (2010–2020).

In addition, we calculated total CH_4_ emissions for our Arctic-Boreal domain using posterior fluxes from the CarbonTracker^26^, CAMS^27^ and from the most recent GCP-CH_4_^1^ inverse models for the same time period (Fig. 6 and Table 3). Note that these models differ in several key aspects, including the atmospheric transport model, model resolution, data assimilated, set of priors optimized, and atmospheric chemistry. At the pan-Arctic scale, our estimates are in better agreement with those of CarbonTracker, although the IAV is smaller in our results than in the CarbonTracker results. This general agreement between models also extends to regional emission patterns. Both our inversion and CarbonTracker estimate mean annual Arctic-Boreal total emissions that are lower than those from CAMS, although their IAV ranges overlap. Furthermore, the regional differences are larger, including lower emissions in Alaska, Canada and Europe, and higher emissions in Russia, as estimated here and by CarbonTracker compared to CAMS (Fig. 6). In comparison with GCP-CH_4_^1^, our Arctic-Boreal estimates are larger (even if we consider the same time period of 2010-2020). Regional differences are also larger, with our model estimating higher emissions in eastern Russia and Alaska, and lower emissions western and eastern Canada, and Europe compared to the GCP-CH_4_ ensembles. These differences may be partly due to the inclusion of regional observation data assimilated in CarboScope and CarbonTracker that are not assimilated in the CAMS and GCP-CH_4_ ensemble inversions.

## Methods

### Model setup

To estimate the CH_4_ emissions between the Arctic-Boreal region and the atmosphere, we use the Jena CarboScope Global Atmospheric Inversion System^21^, which is a linear Bayesian atmospheric inversion framework. This approach optimizes surface-atmosphere fluxes by combining prior flux estimates with atmospheric CH_4_ mole fraction measurements and accounting for their respective uncertainties. The flux vector *f* represents the net flux per grid cell per time step; however, the Jena CarboScope enables *f* to be represented as the sum of different flux components, each of which is modelled independently using its own statistical linear flux model. These components can represent distinct physical sources or processes separated by temporal or spatial scales. Independent a priori error covariance structures enable deviations from the prior flux estimate to be attributed to specific components during the inversion process. Due to the coarse TM3 resolution ( ~ 3.8° x 5°) and overlapping CH_4_ sources and sinks, different processes may contribute together within a grid cell. In such cases, deviations from the prior estimate are partitioned among the components in proportion to their respective uncertainties.

In this study, the a priori shape uncertainty was defined as 100% of the prior flux for each flux category. All flux categories were optimised, assuming a spatial correlation length of ~500 km and a temporal correlation length of about 15 days. Temporal and spatial fluxes are optimized within a Bayesian inversion framework that minimizes a cost function combining prior and observational constraints. The solution is obtained analytically using the linear Bayesian approach^21^, which yields the maximum a posteriori flux estimates and its associated posterior uncertainties. Details of the cost function formulation and solution method can be found in the CarboScope technical report^21^.

Atmospheric tracer transport in the global CarboScope inversions is simulated by The Global Atmospheric Tracer Model TM3^36^ driven by meteorological fields from the NCEP reanalysis^37^. Then, inversions were carried out at a horizontal resolution of ~3.8° latitude and 5° longitude (grid resolution of the transport model TM3), 19 vertical levels, with fluxes having a daily time resolution. Although the original prior fluxes were generally provided in monthly time steps, they were temporally interpolated to generate daily flux fields with smooth variation, ensuring consistency with the temporal resolution required by the inversion system. The total model-data mismatch uncertainty is defined as the sum of the squares of the model and observational components. In line with the standard approach adopted in CarboScope Global^21^, we apply “data density weighting” to balance the influence of discrete (flask) and continuous (e.g. hourly) observations. Without this adjustment, sites with high-frequency data would dominate the cost function simply due to the larger number of observations (N). To avoid this, the individual measurement uncertainty is inflated by $\sqrt{{N}_{{hours}}/{week}}$ where N is the number of observations. This corresponds to the assumption that errors are correlated on weekly timescales, such that one week of hourly data provides roughly the same amount of independent information as one weekly flask sample^21^.

As described by Rödenbeck^21^, the posterior flux uncertainty can be calculated from the prior uncertainties given by the prior flux and the measurement covariance matrices. We calculate the posterior uncertainty for each region in Fig. 1. This uncertainty primarily depends on observation availability and the assumed uncertainties for model-data mismatch and prior fluxes. We reported the annual uncertainties in Tables 1 and 2. The uncertainty reduction (UR; Δσ) is then calculated with: Δσ = 1- σ_Posterior/ σ_Prior). A detailed description of the Jena CarboScope Inversion System is provided in Rödenbeck^21^.

### Atmospheric observations

We assimilated in-situ surface flask and/or continuous CH_4_ measurements from GHG observation sites into the global CarboScope model^21^ over a 12-year period (from 2010 to 2021). CH_4_ observations were obtained from several global and regional networks^38–42^. Data from a total of 154 stations were included (Fig. 1), with the majority located in the Northern Hemisphere, including 33 stations within the Arctic-Boreal region. Most observational data used in this study were accessed through NOAA GML ObsPack^38^, ICOS Carbon Portal^39^, World Data Centre for Greenhouse Gases (WDCGG) database (10.50849/WDCGG_CH4_ALL_2023), and JR-STATION network^41,42^; further details are provided in the “Data Availability section”. Detailed information on the stations with assimilated data is given in Supplementary Table 3. For tower sites with multiple intake heights available, we assimilated only data from the highest height in the inversion, and for the continuous data, we use only daytime measurements. First, a quality check was performed on all CH_4_ data. All assimilated data were in the World Meteorological Organization (WMO) X2004A scale^43^, and for data not originally in this scale, they were then converted by applying specific scale multipliers estimated from WMO/IAEA Round Robin Comparison Experiment (https://gml.noaa.gov/ccgg/wmorr/wmorr_results.php).

### Prior fluxes

Prior fluxes of CH_4_ include five components (wetlands, other natural sources, anthropogenic, ocean and biomass burning emissions here called as fire) derived from available bottom-up models and global inventories (Table 1). Wetland monthly mean emissions between 2010 and 2021 were obtained from the JSBACH model^44^, where wetland CH_4_ emissions are simulated by incorporating surface inundation dynamics alongside CH_4_ production, oxidation, and transport processes. Surface inundation is determined using a TOPMODEL-based approach^45^, which accounts for topographical and hydrological controls. The transport of CH_4_ from sub-surface sources to the atmosphere is represented through diffusion, ebullition, and plant-mediated pathways. This is based on the framework of Riley et al.^46^. A detailed description of JSBACH wetland CH_4_ emission estimates can be found in Kleinen et al.^44^.

Monthly mean CH_4_ emissions from biomass burning (fire) are taken from GCP^1^. Monthly global anthropogenic emissions were obtained from the EDGAR inventories database (https://edgar.jrc.ec.europa.eu) version 8^47^. This component includes emissions from agriculture, livestock, waste, fuel exploitation and other minor anthropogenic sources except biomass burning.

Monthly CH_4_ emissions from other natural sources include natural geological emissions, termites, and herbivory. Geological emission was obtained from Etiope et al.^48^ and includes onshore hydrocarbon macro seeps (including mud volcanoes), submarine (offshore) seeps, diffuse micro seeps, and geothermal manifestations. A non-seasonal climatology (flat-annual) was used as the prior flux. The monthly mean CH_4_ emissions from termites and herbivory were estimated using the JSBACH model^44^. Global ocean emissions were from Weber et al.^49^, which include annual mean emissions from both diffusive and ebullitive processes. A non-seasonal climatology (flat-annual) was used as the prior flux.

### Methane sinks

The global CH_4_ balance is constrained by the magnitude of the emissions from all the different source processes and by the magnitude of its sinks, which are split into the soil uptake and the chemical sinks in the atmosphere. The atmospheric sink includes the CH_4_ oxidation in the troposphere by OH and atomic chlorine (Cl) and its loss in the stratosphere due to reactions with excited atomic oxygen O(^1^D), Cl and OH. In general, most of the inverse models account for CH_4_ oxidation by OH and O(^1^D), and some include stratospheric Cl oxidation but not the tropospheric oxidation. In this study, the chemical loss includes all these components: loss due to OH and Cl in the troposphere, and due to OH, Cl and O(^1^D) in the stratosphere, which is a similar setup for chemical loss as used by NOAA's CarbonTracker, version CT-CH4-2023^26,50^. For tropospheric OH, we use the monthly three-dimensional OH fields calculated by Spivakovsky et al.^51^ based on observed climatological distributions of OH precursors and scaled to match the observed CH_3_CCl_3_ lifetime. Monthly climatological CH_4_ loss rates in the stratosphere due to OH, Cl, and O(^1^D) were constructed from a run of the ECHAM5/MESSy1 chemistry transport model^52^, and the loss due to tropospheric Cl is simulated using a recent model-derived estimate of tropospheric Cl^53^.

In addition to the chemical sink in the atmosphere, the surface sink from upland soils and oceans was also included. In this study, CH_4_ sinks are prescribed rather than optimized in the inversion and without IAV. While the majority of inverse models include the surface uptake of atmospheric CH_4_ as part of the net surface fluxes, here it was implemented as a zeroth order reaction with prescribed reaction rates occurring only in the model layer closest to the surface. The reaction rates for microbial soil oxidation of atmospheric CH_4_ were based on the uptake estimates of the LPJ-Bern model^54^ within the context of the WETCHIMP experiment 2. The total CH_4_ loss for the period between 2010 to 2021 reported here is 595.9 TgCH_4_ y^−1^, where the chemical sink represents 93% of the total sink. The total lifetime of CH_4_ over this time is 8.6 years, which is similar to previous estimates (an average of 8.2 years over 2000–2009, accounting for soil uptake and total chemical loss, with a range of 6.8–9.7 years^1^).

### Design of sensitivity tests

To evaluate the effect of the addition of regional data on estimated CH_4_ budgets, we have also performed an inversion using only seven stations within the Arctic-Boreal domain (Supplementary Fig. 11), while retaining all other stations assimilated in the reference run outside of this domain. These seven stations are generally considered background stations, as they have only limited influence from local sources. For simplicity, we refer to the posterior emissions from the inversion using only data from the seven stations within our domain as “posterior emissions (BKG stations)” and from the inversion with all the other regional data assimilated as “posterior emissions”, respectively.

To evaluate the influence of prior IAV on posterior wetland CH_4_ flux estimates, we performed an additional inversion using a climatological wetland prior that kept the mean seasonal cycle but removed the IAV.

In the CarboScope system, a factor μ is defined to control the relative weighting between a-priori information and observational data^21^. Although atmospheric observation availability in the Arctic has improved over the past decade, large spatial gaps with regions with limited coverage persist. To compensate for this irregular distribution of data, we adopted *μ* = 5, in our reference setup, which corresponds to scaling the a-priori uncertainty by 1/√5. This choice helps prevent the inversion from producing unrealistic posterior fluxes in data-sparse areas. To assess the sensitivity of the posterior estimates to this choice, we conducted three additional experiments: one with μ = 1, representing no scaling of the prior uncertainty, a second one with *μ* = 30 (scaling by 1/√30) and another one with μ = 60, corresponding to stronger prior influence (scaling by 1/√60). This range of *μ* values allows us to evaluate how robust the inversion results are to changes in the relative strength of prior constraints. We used the results of four sensitivity tests with the same set of stations as our reference run (wetland prior as climatology, *μ* = 1, *μ* = 30 and *μ* = 60) to estimate a range of posterior estimates.

### Evaluating the model with independent observations

To validate our inversion results, we used independent in situ observations of CH_4_ mole fractions^38,39,42^ in the Arctic-Boreal region. These were taken at the Birkenes Observatory (BIR; 58.3886^∘^ N, 8.2519^∘^ E) in Norway between 2010 and 2011, using the highest tower sampling level (75 m). We also used measurements taken at Chibougamau (CHM; 49.6925^∘^ N, 74.3432^∘^ W) in Canada, between 2010 and 2011; at Station Nord (SNO; 81.3605^∘^ N, 16.3943^∘^ W) in Greenland between 2019 and 2021, and at Yakutsk (YAK; 62.0886^∘^ N, 129.3558^∘^ W), Russia, which we used measurements taken at 77 m height, between 2010 and 2011. These data meet the highest quality standards and are also part of global atmospheric monitoring networks^38,39^. However, they were excluded from the main inversion analysis due to the short period of their data records. To compare modelled and observed mole fractions, we sampled the model data at the grid cell closest to the site locations, for the closest hour and altitude. We then calculated the mean bias (model - observation) and the correlation coefficients between modelled and observed CH_4_ mole fractions for all the four stations.

### Analysis of climate drivers of CH_4_ emissions

To investigate CH_4_ emissions drivers we compared our prior and posterior CH_4_ fluxes to the spatial-temporal patterns of meteorological data such as total precipitation, air temperature and snow depth, with data grids taken from the ERA5 global reanalysis^55^. The ERA5 data used is a monthly averaged data with a spatial resolution of 0.25° × 0.25°, available for the period 1980 to 2021. Total precipitation is the accumulated liquid and frozen water, comprising rain and snow, that falls to the Earth's surface. Air temperature is the temperature of air at 2 m above the surface of land, sea or inland waters. The snow depth is the amount of snow from the snow-covered area of a grid box, and it is the depth the water would have if the snow melted and was spread evenly over the whole grid box.

To assess the relationship between CH_4_ fluxes and climate drivers, we performed both linear and multiple linear regression analyses using air temperature, total precipitation, and snow depth as predictors. The analysis was done using annual mean values, as well as separately for specific periods, for example warm period (mean of May-October for each year). Model performance was evaluated using the Akaike Information Criterion–AIC^28^ to determine whether including additional variables improved the explanatory power of the multiple regression models.

To further investigate CH_4_ emission dynamics, considering that IAV in CH_4_ emissions across the Arctic-Boreal region is not spatially homogeneous, we analysed CH_4_ emission trends and their climate drivers for each sub-region. Given the large spatial scale of these regions, localised trends in specific hotspot areas may be smoothed out and not fully captured in the regional analysis. To address this, we conducted a more detailed analysis at the grid-cell level, examining trends on a monthly and annual basis to identify finer-scale variations in emission patterns. The climate data were reprojected to match the spatial resolution of the posterior flux estimates before performing the grid-cell level correlation analysis.

## Supplementary information

Supplementary Figure

## Data Availability

The posterior mean Arctic-Boreal CH4 fluxes on the CarboScope model horizontal resolution are available for download at Zenodo (10.5281/zenodo.18563123). Observations from the NOAA GML network can be downloaded from the dedicated Observation Package (ObsPack) web server at 10.25925/20231001. The dataset “European atmospheric CO_2_, CH_4_ and N_2_O Mole Fraction data product – 2024” analysed during the current study was downloaded from the ICOS Carbon portal, DOI: 10.18160/KDMT-V6CG. The observations from the WDCGG dataset are available at 10.50849/WDCGG_CH4_ALL_2023. ATTO tower data can be request at https://www.attodata.org/. Observations from the Japan-Russia Siberian Tall Tower Inland Observation Network (JR-STATION)^42^ can be downloaded from 10.17595/20231117.001, 10.17595/20231117.002, 10.17595/20231117.004, 10.17595/20231117.005, 10.17595/20231117.006, 10.17595/20231117.007, 10.17595/20231117.008. CH_4_ observations from the western Taimyr Peninsula (Siberia) can be downloaded from 10.17632/gcts3dddrh.1.
