# Supplementary material for: Quantifying Arctic-boreal methane emissions using atmospheric observations and a global inverse model
Source: NPJ Clim Atmos Sci. 2026 Feb 14;9(1):80. doi: 10.1038/s41612-026-01348-1 (PMC13043286; doi:10.1038/s41612-026-01348-1)
Supplement: Supplementary file 1 — Supplementary Figure [file 41612_2026_1348_MOESM1_ESM.pdf]

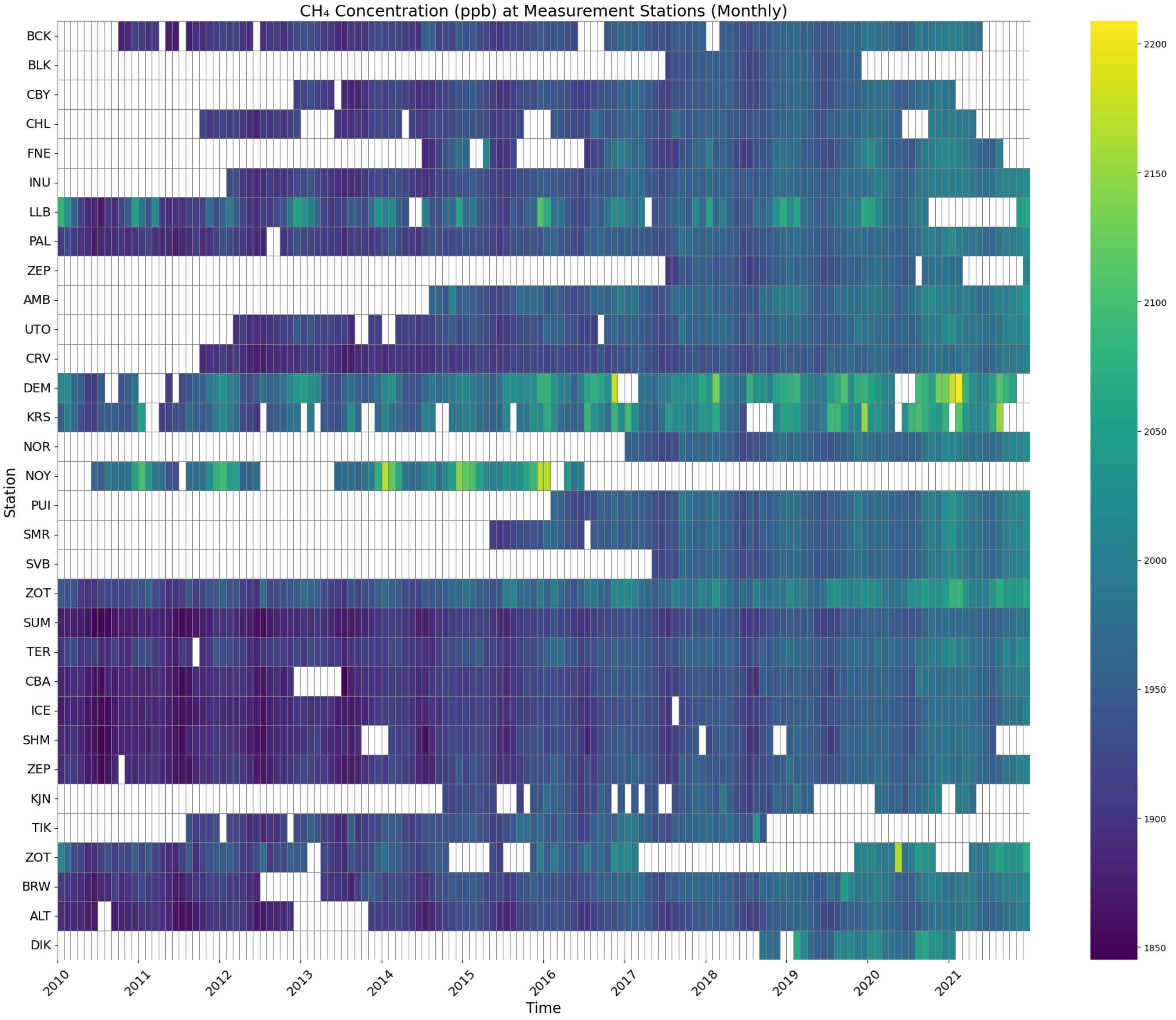

Supplementary Figure 1. Monthly mean CH_4 mixing ratios at stations in the Arctic-Boreal region with data assimilated in the inversion system between 2010 and 2021.

Supplementary Table 1. Prior and posterior model bias (ppb) and correlation between CarboScope and Arctic-Boreal observations from assimilated sites.

Station	Prior mean bias (ppb)	Posterior mean bias (ppb)	Prior correlation (r^2)	Posterior correlation (r^2)
ALT	-80.75	2.26	0.70	0.95
AMB	-111.50	-3.92	0.18	0.45
BCK	-95.73	1.50	0.11	0.65
BLK	-108.29	0.78	0.18	0.72
BRW	-77.60	-0.97	0.33	0.71
CBA	-86.95	-4.40	0.62	0.91
CBY	-101.21	0.03	0.59	0.85
CHL	-101.46	-0.50	0.32	0.75
CPS	-98.29	0.56	0.29	0.85
CRV	-92.16	4.06	0.36	0.74
DEM	-150.88	-28.64	0.41	0.56
DIK	-124.44	-6.23	0.29	0.61
ETL	-110.07	-7.88	0.44	0.63
FNE	-128.39	-13.05	0.20	0.41
FSD	-85.27	-2.55	0.40	0.85
ICE	-80.50	0.10	0.62	0.94
INU	-113.49	-14.79	0.27	0.67
KJN	-103.05	3.79	0.65	0.86
KRS	-131.83	-12.30	0.47	0.64
LLB	-139.12	-24.94	0.22	0.42
NOR	-108.96	-0.22	0.55	0.78
NOY	-153.78	-18.74	0.28	0.47
PAL	-87.17	-1.86	0.71	0.87
PUI	-103.91	2.55	0.54	0.79
SHM	-85.75	-2.83	0.64	0.93
SMR	-99.79	3.01	0.55	0.83
SUM	-82.54	2.64	0.70	0.96
SVB	-105.74	2.11	0.60	0.82
TER	-85.19	-4.50	0.58	0.89

TIK	-99.10	-3.69	0.14	0.53
UTO	-99.69	0.39	0.56	0.82
ZEP	-102.98	5.47	0.67	0.92
ZOT	-113.64	1.92	0.42	0.64

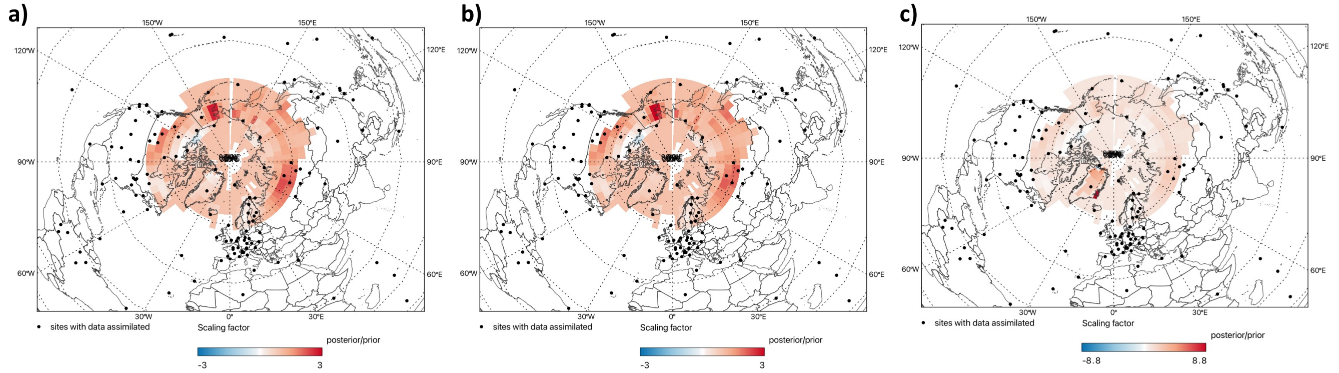

Supplementary Figure 2. Scaling factor of a) annual mean fluxes from 2010 to 2021 (annual mean posterior fluxes / annual mean prior fluxes), b) 2010 annual mean flux, and c) 2021 annual mean flux. Note the different color scale in panel c. Positive values indicate regions where prior estimates underestimated emissions compared with posterior estimates, while negative values represent areas where prior emissions overestimate CH_4 emissions compared with the posterior estimates.

Supplementary Table 2. Prior and posterior model bias (ppb) and correlation between CarboScope and independent Arctic-Boreal observations from non-assimilated sites.

**Station**	**Prior mean bias (ppb)**	**Posterior mean bias (ppb)**	**Prior correlation (r^2)**	**Posterior correlation (r^2)**
BIR	-117.35	4.41	0.42	0.59
CHM	-84.08	2.83	0.03	0.45
SNO	-114.97	4.77	0.26	0.64
YAK	-99.19	-6.37	0.08	0.11

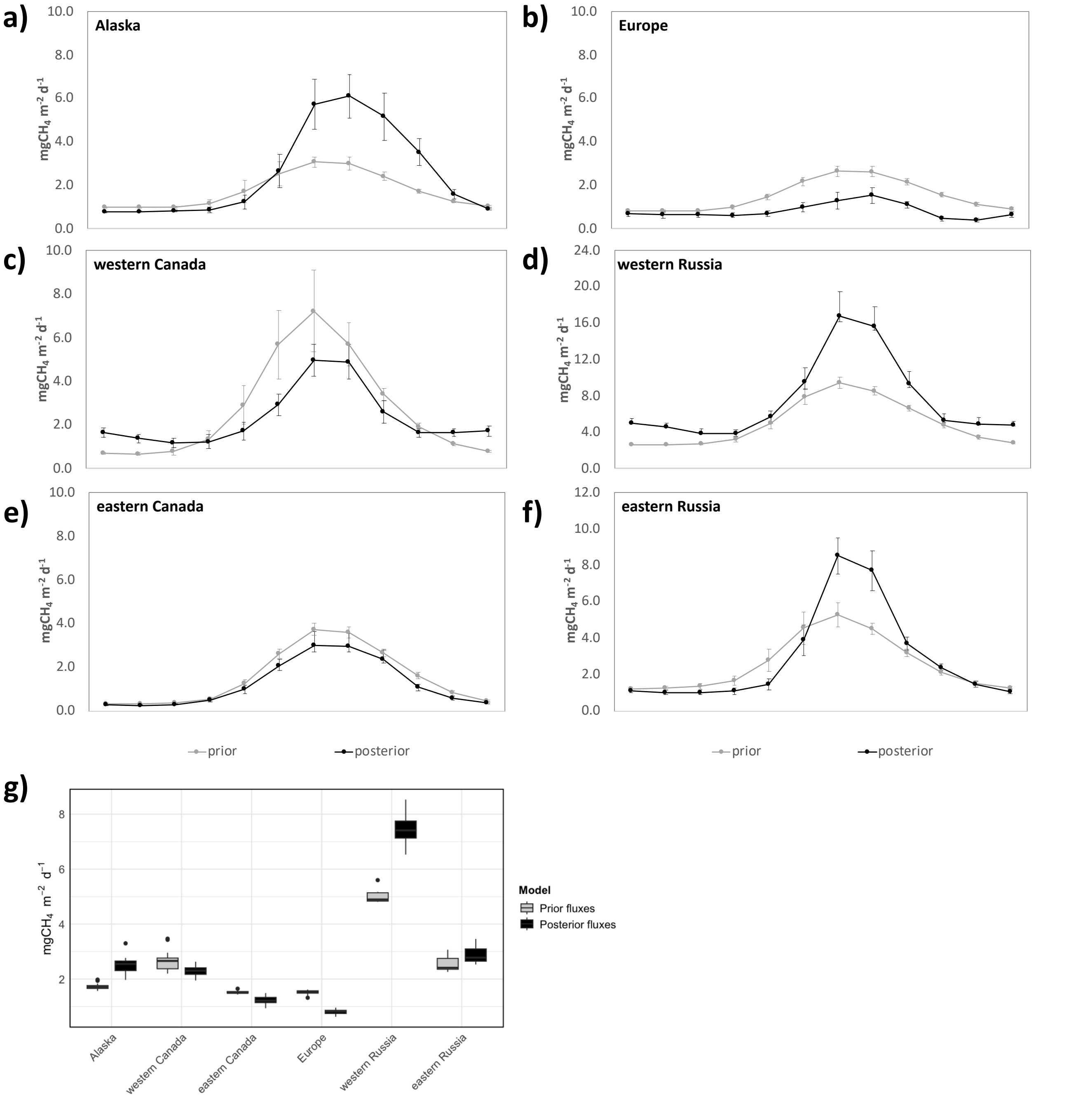

Supplementary Figure 3. (a-f) The 12-year monthly mean (2010-2021) total land CH_4 prior and posterior fluxes (excluding ocean emissions; grid cells with both land and ocean areas are included) for each of the sub-regions. Note the different scale range for western Russia. Error bars represent one standard deviation for each month over the 12-year period (one sigma). (g) the annual mean of total prior and posterior fluxes for each of the sub-regions (excluding exclusively ocean grid-cells).

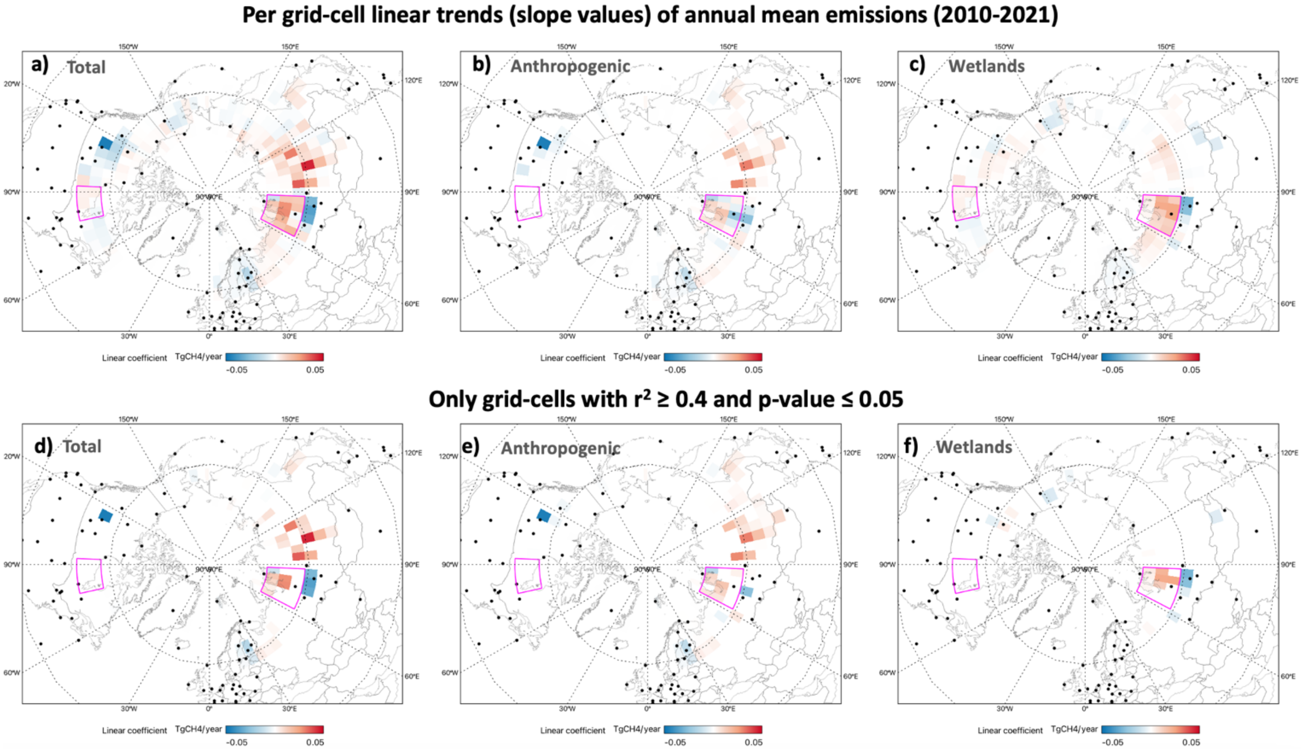

Supplementary Figure 4. Per grid-cell linear trends (slope values) of posterior annual mean CH_4 emissions from 2010 to 2021 across the Arctic-Boreal domain. Panels (a-c) show slope values for (a) total, (b) anthropogenic, and (c) wetland posterior CH_4 emissions, with all grid-cells shown regardless of statistical significance. Panels (d-f) show only grid-cells with statistically significant trends ($r^2 \geq 0.4$ and $p \leq 0.05$) for (d) total, (e) anthropogenic, and (f) wetland posterior CH_4 emissions. Positive slope values indicate increasing CH_4 emissions over time, while negative values indicate decreasing emissions. Pink contours delineate the two CH_4 hotspot areas, the Western Siberia Lowlands (WSL) and the Hudson Bay Lowlands (HBL), and black dots represent the geographic distribution of surface sites where flask-based and/or continuous in-situ CH_4 measurements are assimilated in the inverse model.

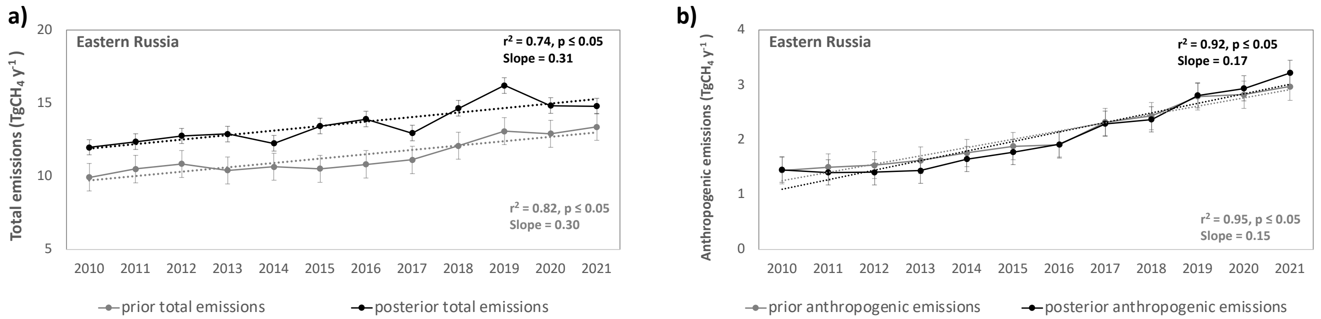

Supplementary Figure 5. The annual mean CH_4 emissions for the eastern Russia (2010–2021) are displayed as (a) total emissions and (b) anthropogenic emissions. Error bars represent the prior and posterior annual uncertainties calculated as described in Methods section "Model setup". Each panel shows the prior (grey line) and posterior (black line) estimates of emissions, along with their respective linear regression trends over time.

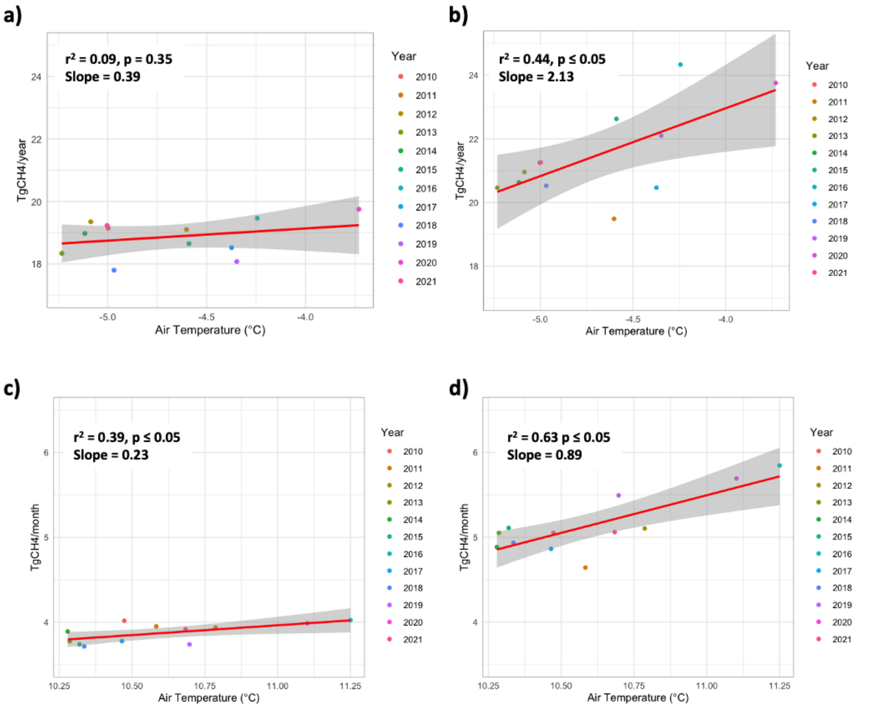

Supplementary Figure 6. Linear correlation between annual mean wetland (a) prior and (b) posterior flux and annual mean air temperature in the Arctic-Boreal region, and linear correlation between mean (July-September) wetland (c) prior and (d) posterior flux and mean (July-September) air temperature in the Arctic-Boreal region.

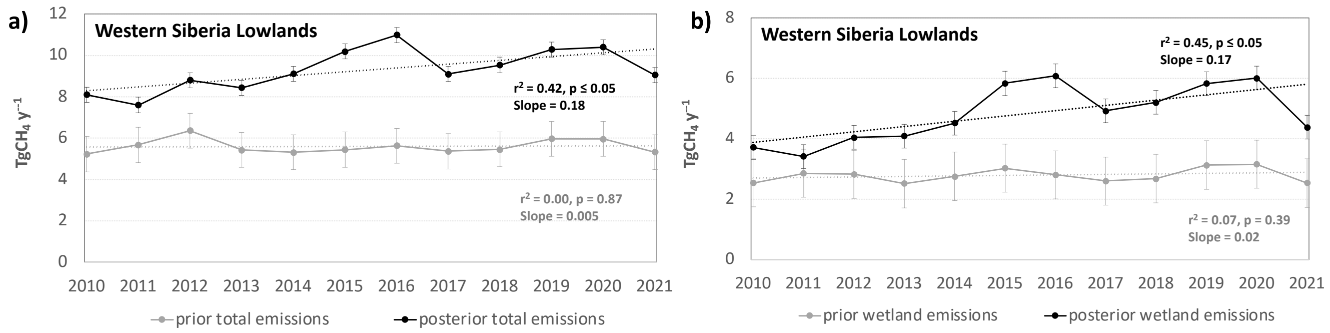

Supplementary Figure 7. The annual mean CH_4 emissions for the Western Siberia Lowlands (2010–2021) are displayed as total emissions (a) and wetland emissions (b). Error bars represent the prior and posterior annual uncertainties calculated as described in Methods section “Model setup”. Each panel shows the prior (grey line) and posterior (black line) estimates of emissions, along with their respective linear regression trends over time.

Per grid-cell linear trends (slope values and r^2) of July-September mean wetland posterior mean emissions and air temperature

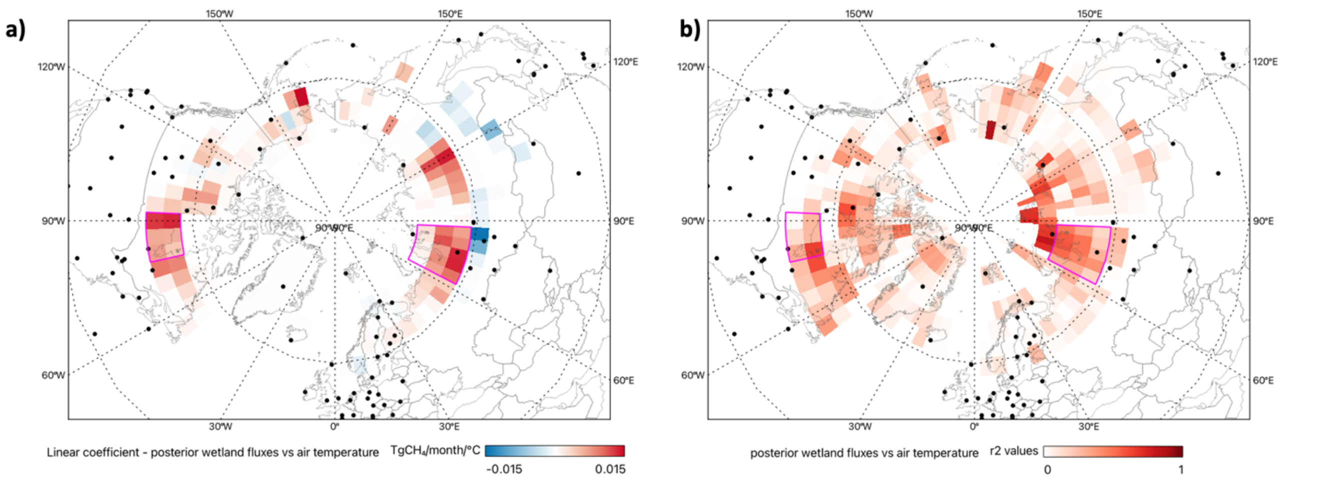

Supplementary Figure 8. Per grid-cell linear trends (slope values) of posterior late growing season (July-September) mean CH_4 emissions from 2010 to 2021 and air temperature across the Arctic-Boreal domain. Panel (a) show slope values and panel (b) the r^2 value. Pink contours delineate the two CH_4 hotspot areas, the Western Siberia Lowlands (WSL) and the Hudson Bay Lowlands (HBL), and black dots represent the geographic distribution of surface sites where flask-based and/or continuous in-situ CH_4 measurements are assimilated in the inverse model.

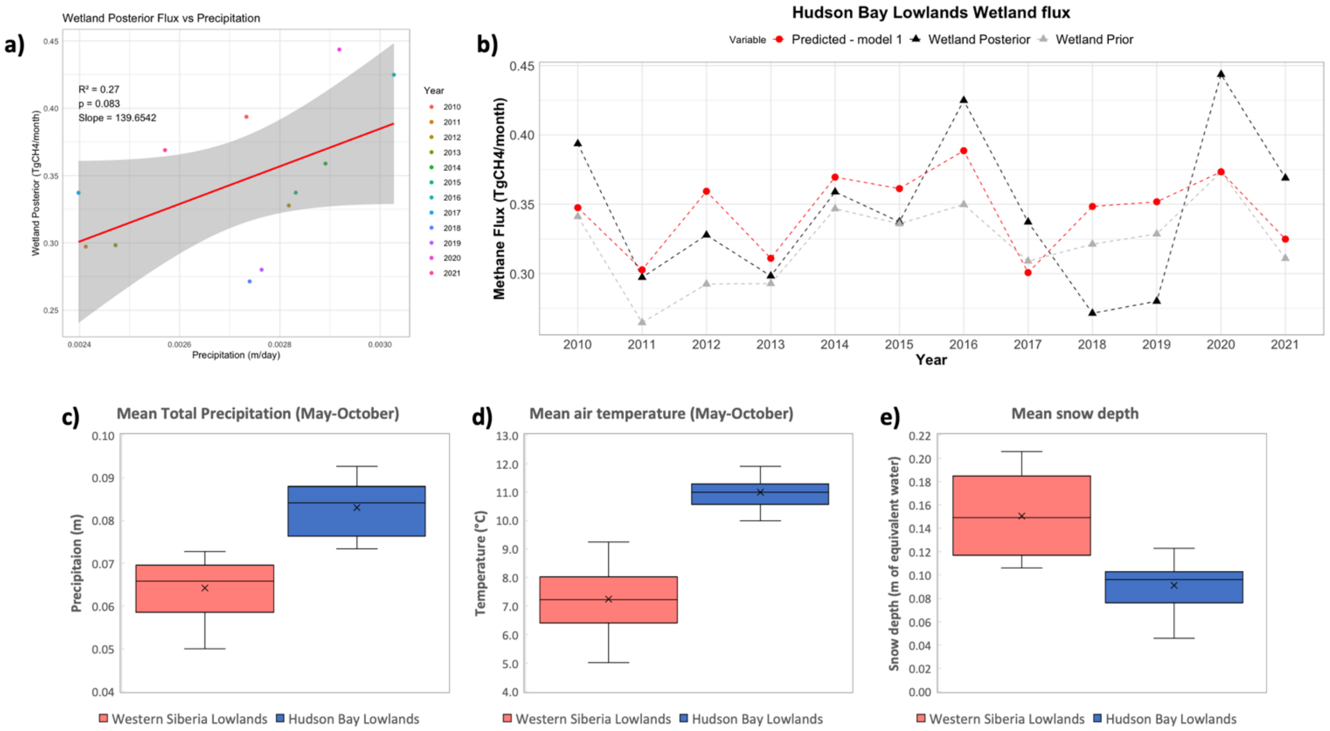

Supplementary Figure 9. a) Linear correlation between mean wetland posterior flux and mean total precipitation in the Hudson Bay Lowlands (HBL) between May and October. (b) Mean CH_4 wetland emissions during the warm period (May-October) for the HBL are displayed as posterior (black) and prior (grey) emissions, and predicted emissions using a linear regression with mean total precipitation (May-October; named model 1, red). Box-plots of (c) mean total precipitation and (d) mean air temperature during the growing season (May-October), and (e) mean snow depth (March-April for Western Siberia Lowlands and February-March for Hudson Bay Lowlands).

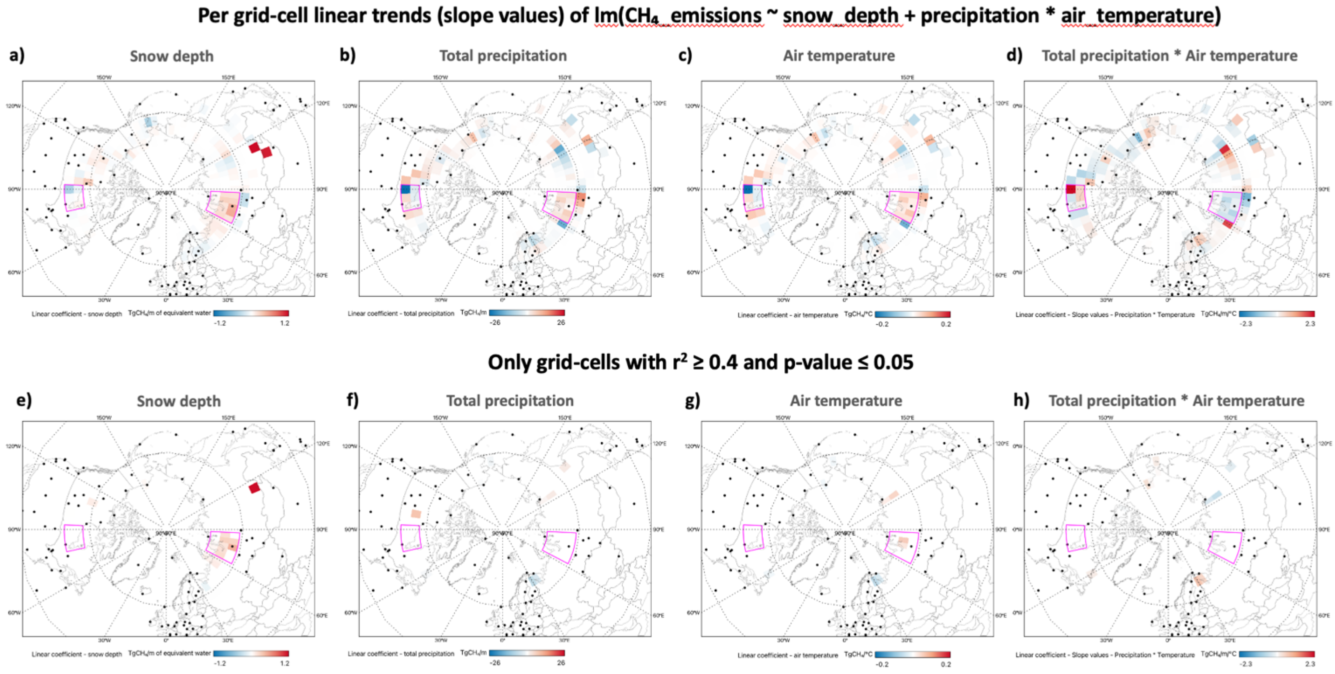

Supplementary Figure 10. Per grid-cell slope values from a multilinear regression model relating mean CH_4 emissions during the growing season (May-October) to environmental drivers across the Arctic-Boreal domain from 2010 to 2021. The model includes mean snow depth during late-winter (March-April), and growing season (May-October) mean air temperature and total precipitation, including their interaction: CH_4_emissions ~ snow_depth + precipitation * air_temperature. Panels (a-d) show slope values for (a) snow depth, (b) total precipitation, (c) air temperature, and (d) the interaction of total precipitation and air temperature, with all grid-cells shown regardless of statistical significance. Panels (e-h) show only grid-cells with statistically significant trends ($r^2 \geq 0.4$ and $p \leq 0.05$) for (e) snow depth, (f) total precipitation, (f) air temperature, and (g) total precipitation and air temperature interaction. Positive slope values indicate an increase in CH_4 emissions with increasing predictor values, while negative values indicate a decrease. Pink contours delineate the two CH_4 hotspot areas, the Western Siberia Lowlands (WSL) and the Hudson Bay Lowlands (HBL), and black dots represent the geographic distribution of surface sites where flask-based and/or continuous in-situ CH_4 measurements are assimilated in the inverse model.

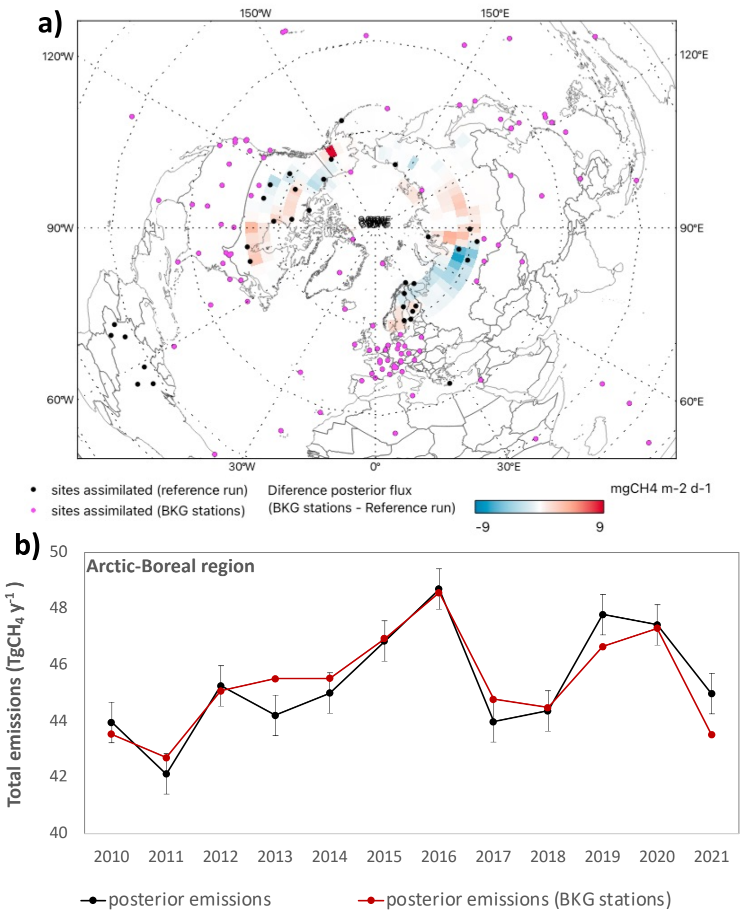

Supplementary Figure 11. a) Annual mean (2010-2021) difference between posterior total flux reference run (assimilating the regional stations data; black dots) and total posterior flux from the inversion assimilating only background (BKG) stations (pink dots) inside our Arctic-Boreal domain. Positive values indicate regions where inversion without assimilating stations represented by black dots overestimates methane emissions compared to the reference run. And negative values represent regions where inversion without assimilating stations represented by black dots underestimates methane emissions compared to the reference run. b) Time series of annual mean posterior total flux reference run (assimilating the regional stations data; black dots) and total posterior flux from the inversion assimilating only background (BKG) stations (pink dots) inside our Arctic-Boreal domain. Error bars represent the posterior annual uncertainties calculated as described in Methods section "Model setup".

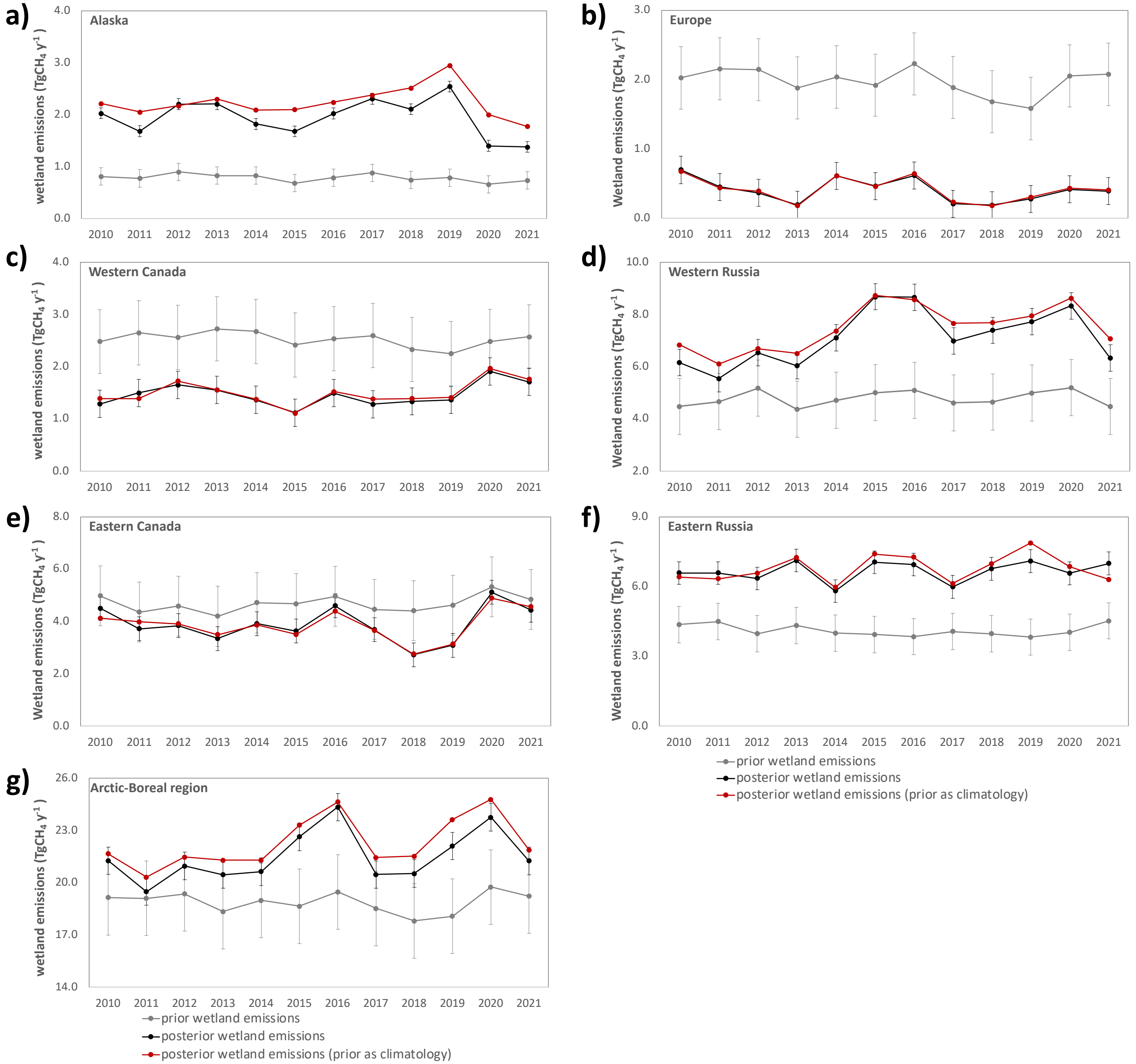

Supplementary Figure 12. (a-f) Time series of annual mean posterior wetland fluxes from the reference run (posterior wetland emissions), the posterior fluxes from the inversion using a climatological wetland prior (without interannual variability, IAV), and the wetland prior fluxes (with IAV) for each sub-region; (g) same as before, but for the entire Arctic-Boreal domain. Error bars represent the prior and posterior annual uncertainties calculated as described in Methods section "Model setup".

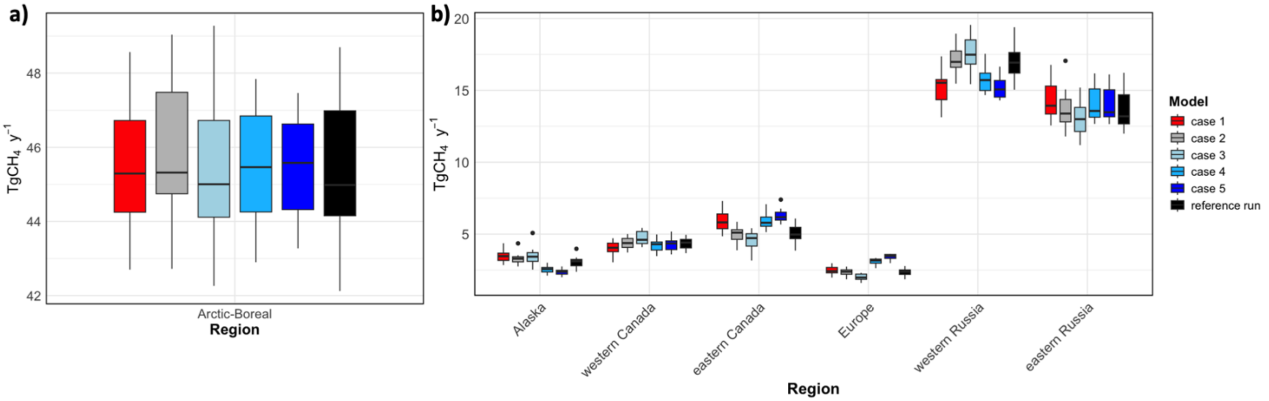

Supplementary Figure 13. Boxplot of annual mean total emissions at (a) Arctic-Boreal region and (b) per each sub-region for our reference run and each one of the sensitivity tests as: case 1 - posterior emissions from the inversion using only data from the seven stations within our domain; case 2 - inversion using a climatological wetland prior (without IAV); case 3 – posterior fluxes based on $\mu = 1$, representing larger prior uncertainty than in the reference run; case 4 – posterior fluxes based on $\mu = 30$, corresponding to the prior uncertainty (scaling by $1/\sqrt{30}$); and case 5 – posterior fluxes based on $\mu = 60$, corresponding to the prior uncertainty (scaling by $1/\sqrt{60}$).

Supplementary Table 3. Details of monitoring sites with CH_4 observations data used in the inverse model.

Code	Latitude	Longitude	Time-period
ABT	49.01° N	122.34° W	2014 - 2020
ALF	8.92° S	56.79° W	2010 -2018
ALT	82.45° N	62.50° W	2000 - 2021
AMB	69.62° N	162.30° E	2014 - 2021
AMT	45.03° N	68.68° W	2008 - 2021
AMY	36.54° N	126.33° E	2013 - 2021
ARH	77.83° S	162.20° E	2000 - 2021
ASC	7.97° S	14.40° W	2000 - 2021
ASK	23.26° N	5.63° E	2000 - 2021
ATT	2.14° S	58.99° W	2012 -2019
AZR	38.76° N	27.29° W	2000 - 2021
AZV	54.71° N	73.03° W	2009 - 2019
BCK	62.8° N	115.92° W	2010 - 2021
BHD	41.41° S	174.87° E	2000 - 2021
BIK	53.22° N	23.01° E	2005 - 2021
BIS	44.38° N	1.23° W	2009 - 2021
BKT	0.20° S	100.32° E	2004 - 2021
BLK	64.33° N	96.01° W	2017 - 2019
BMW	32.26° N	64.88º W	2000 - 2021

BRA	50.20° N	104.71º W	2009 - 2021
BRW	71.34° N	156.64° W	2000 - 2021
BRZ	56.15° N	84.33° E	2008 - 2019
CBA	55.21° N	162.72° W	2000 - 2021
CBW	51.97° N	4.93° E	2000 - 2020
CBY	69.13° N	105.06° W	2012 - 2021
CFA	19.28° N	147.06° E	2000 - 2021
CGO	40.68° S	144.69° E	2000 - 2021
CHL	58.74° N	93.82° W	2011 - 2021
CHR	1.70° N	157.17° W	2000 - 2020
CIB	41.81° N	4.93° W	2009 - 2021
CMN	44.19° N	10.70° E	2008 - 2021
COI	43.16° N	145.50° E	1995 - 2021
CPA	42.64° N	77.07° E	2016 - 2021
CPS	49.82° N	74.98° W	2011 - 2021
CPT	34.35° S	18.49° E	2000 - 2021
CRV	64.99° N	147.60° W	2011 - 2021
CRZ	46.43° S	51.85° E	2000 - 2021
CVO	16.86° N	24.87° W	2007 - 2021
CYA	66.28° S	110.52° E	2000 - 2021
DEM	59.79° N	10.87° E	2005 - 2019
DIK	73.51° N	80.52° E	2018 - 2021
DRP	59.16° S	62.59° W	2006 - 2021
DSI	20.69° N	116.73° E	2010 - 2021
EGB	44.23° N	79.78° W	2005 - 2021
EIC	27.15° S	109.45° W	2000 - 2019
ESP	49.38° N	126.54° W	2009 - 2021
EST	51.67° N	110.21° W	2010 - 2021
ETL	54.35° N	104.99° W	2005 - 2021
FNE	58.84° N	122.57° W	2014 - 2021
FSD	49.88° N	81.57° W	2000 - 2021
GAT	53.07° N	11.44° E	2016 - 2021
GMI	13.39° N	144.66° E	2000 - 2021
GPA	12.25° S	131.04° E	2010 - 2021
HAT	24.06° N	123.81° E	1996 - 2021
HBA	75.61° S	26.21° W	2000 - 2021
HNP	43.61° N	79.39° W	2014 - 2021
HPB	47.80° N	11.02° E	2006 - 2021
HSU	41.05° N	124.60° W	2008 - 2017
HTM	56.10° N	13.42° E	2016 - 2021
HUN	46.95° N	16.65° W	2000 - 2021

ICE	63.39° N	20.29° W	2000 - 2021
INU	68.32° N	133.53° W	2012 - 2021
IPR	45.81° N	8.63° E	2017 - 2021
IZO	28.31° N	16.49° W	2000 - 2021
JFJ	46.55° N	7.98° E	2007 - 2021
KAS	49.23° N	19.98° E	2000 - 2021
KEY	25.61° N	80.20° W	2000 - 2021
KJN	70.85° N	29.22° E	2014 - 2021
KRE	49.58° N	15.08° E	2017 - 2021
KRS	58.25° N	82.42° E	2004 - 2019
KUM	19.52° N	154.82° W	2000 - 2021
LAU	45.03° S	169.67° E	2009 - 2021
LEF	45.95° N	90.27° W	2010 - 2021
LEW	40.94° N	76.88° W	2013 - 2021
LIN	52.17° N	14.12° E	2015 - 2021
LLB	54.95° N	112.45° W	2007 - 2021
LLN	23.46° N	120.86° E	2006 - 2021
LMP	35.52° N	12.63° E	2008 - 2021
LUT	53.40° N	6.35° E	2006 - 2021
MAA	67.62° S	62.87° E	2000 - 2021
MBO	43.98° N	121.69° W	2011 - 2021
MEX	18.98° N	97.31° W	2009 - 2021
MHD	53.33° N	9.89° W	2000 - 2021
MID	28.22° N	177.37° W	2000 - 2021
MKN	0.06° S	37.30° E	2003 - 2011
MLO	19.53° N	155.58° W	2000 - 2021
MNM	24.29° N	153.98° E	2000 - 2021
MQA	54.48° S	158.97° E	2000 - 2021
MSH	41.66° N	70.50° W	2016 - 2021
MWO	34.22° N	118.06° W	2010 - 2021
NAT	5.51° S	35.26° W	2010 - 2020
NMB	23.58° S	15.03° E	2000 - 2021
NOR	60.09° N	17.48° E	2017 - 2021
NOY	63.43° N	75.78° E	2005 - 2019
NWP	4.09° S	152.2° E	2000 - 2017
NWR	40.05° N	105.63° W	2000 - 2021
OHP	43.92° N	5.75° E	2014 - 2021
OPE	48.56° N	5.5° E	2011 - 2021
OXK	50.03° N	11.81° E	2003 - 2021
PAL	67.97° N	24.12° E	2004 - 2021
PDI	21.57° N	103.52° E	2014 - 2020

PDM	42.94° N	0.14° E	2014 - 2021
POC	1.31° S	114.59° W	2000 - 2017
PRS	45.93° N	7.70° W	2005 - 2021
PSA	64.77° S	64.05° W	2000 - 2021
PUI	62.91° N	27.65° E	2016 - 2021
PUY	45.77° N	2.97° E	2011 - 2021
RBA	9.36° S	67.62° W	2010 -2018
RGL	52.00° N	2.54° W	2012 - 2021
RPB	13.16° N	59.43° W	2000 - 2021
RUN	21.08° S	55.38° E	2018 - 2021
RYO	39.03° N	141.82° E	2000 - 2021
SAC	48.72° N	2.14° E	2015 - 2021
SAN	2.85° S	54.95° W	2004 - 2018
SCT	33.41° N	81.83° W	2015 - 2021
SEY	4.68° S	55.53° E	2000 - 2021
SGP	36.61° N	97.49° W	2002 - 2021
SHM	52.72° N	174.12° E	2000 - 2021
SIS	59.85° N	1.27° W	2003 - 2021
SMO	14.24° S	170.56° W	2000 - 2021
SMR	61.85° N	24.29° E	2015 - 2021
SPO	89.98° S	24.80° W	2000 - 2021
STR	37.76° N	122.45° W	2007 - 2014
SUM	72.59° N	38.42° W	2000 - 2021
SVB	64.26° N	19.77° E	2017 - 2021
SVV	51.33° N	82.13° E	2007 - 2014
SYO	69.01° S	39.58° E	2000 - 2021
TAB	5.98° S	69.70° W	2010 - 2012
TAC	52.52° N	1.14° E	2013 - 2021
TAP	36.73° N	126.13° E	2000 - 2021
TEF	3.70° S	66.50° W	2013 - 2018
TER	69.20° N	35.10° E	2000 - 2021
THD	41.05° N	124.15° W	2002 - 2017
TIK	71.59° N	128.89° E	2011 - 2018
TLL	30.17° S	70.80° W	2013 - 2021
TOH	51.81° N	10.53° E	2017 - 2021
TPD	42.64° N	80.56° W	2012 - 2021
TRN	47.96° N	2.11° E	2007 - 2021
ULD	37.48° N	130.9° E	2014 - 2021
USH	54.85° S	68.31° W	2000 - 2021
UTA	39.90° N	113.72° W	2000 - 2021
UTO	59.78° N	21.37° E	2012 - 2021

UUM	44.45° N	111.10° E	2000 - 2021
VGN	54.50° N	62.32° E	2008 - 2019
WBI	41.72° N	91.35° W	2007 - 2021
WGC	38.26° N	121.49° W	2007 - 2021
WIS	30.86° N	34.78° E	2000 - 2021
WKT	31.31° N	97.33° W	2001 - 2021
WLG	36.27° N	100.92° E	2000 - 2021
WSA	43.93° N	60.01° W	2003 - 2021
YON	24.47° N	123.01° E	2000 - 2021
ZEP	78.91° N	11.89° E	2000 - 2021
ZOT	60.80° N	89.35° E	2006 - 2021
ZSF	47.42° N	10.98° E	2002 - 2021
